# Antimicrobial resistance (AMR) in COVID-19 patients: a systematic review and meta-analysis (November 2019–June 2021)

**DOI:** 10.1186/s13756-022-01085-z

**Published:** 2022-03-07

**Authors:** Ruwandi M. Kariyawasam, Danielle A. Julien, Dana C. Jelinski, Samantha L. Larose, Elissa Rennert-May, John M. Conly, Tanis C. Dingle, Justin Z. Chen, Gregory J. Tyrrell, Paul E. Ronksley, Herman W. Barkema

**Affiliations:** 1 Antimicrobial Resistance - One Health Consortium, Calgary, AB Canada; 2 Division of Diagnostic and Applied Microbiology, Department of Laboratory Medicine and Pathology, Faculty of Medicine and Dentistry, University of Alberta, Edmonton, AB Canada; 3 Alberta Precision Laboratories - Public Health Laboratory (ProvLab), Edmonton, AB Canada; 4 Departments of Medicine, Microbiology, Immunology and Infectious Diseases, and Community Health Sciences, O’Brien Institute for Public Health and Snyder Institute for Chronic Diseases, University of Calgary, Calgary, AB Canada; 5 Departments of Medicine, Pathology and Laboratory Medicine, Microbiology, Immunology and Infectious Diseases, O’Brien Institute for Public Health, Snyder Institute for Chronic Diseases, University of Calgary and Alberta Health Services, Calgary, AB Canada; 6 Division of Infectious Diseases, Department of Medicine, Faculty of Medicine and Dentistry, University of Alberta, Edmonton, AB Canada; 7 Department of Community Health Sciences, O’Brien Institute for Public Health, University of Calgary, Calgary, AB Canada; 8 Departments of Production Animal Health and Community Health Sciences,, One Health at UCalgary, University of Calgary, 3330 Hospital Drive NW, Calgary, T2N 4N1 Canada

**Keywords:** Antimicrobial resistance, COVID-19, SARS-CoV-2

## Abstract

**Background:**

Pneumonia from SARS-CoV-2 is difficult to distinguish from other viral and bacterial etiologies. Broad-spectrum antimicrobials are frequently prescribed to patients hospitalized with COVID-19 which potentially acts as a catalyst for the development of antimicrobial resistance (AMR).

**Objectives:**

We conducted a systematic review and meta-analysis during the first 18 months of the pandemic to quantify the prevalence and types of resistant co-infecting organisms in patients with COVID-19 and explore differences across hospital and geographic settings.

**Methods:**

We searched MEDLINE, Embase, Web of Science (BioSIS), and Scopus from November 1, 2019 to May 28, 2021 to identify relevant articles pertaining to resistant co-infections in patients with laboratory confirmed SARS-CoV-2. Patient- and study-level analyses were conducted. We calculated pooled prevalence estimates of co-infection with resistant bacterial or fungal organisms using random effects models. Stratified meta-analysis by hospital and geographic setting was also performed to elucidate any differences.

**Results:**

Of 1331 articles identified, 38 met inclusion criteria. A total of 1959 unique isolates were identified with 29% (569) resistant organisms identified. Co-infection with resistant bacterial or fungal organisms ranged from 0.2 to 100% among included studies. Pooled prevalence of co-infection with resistant bacterial and fungal organisms was 24% (95% CI 8–40%; n = 25 studies: I^2^ = 99%) and 0.3% (95% CI 0.1–0.6%; n = 8 studies: I^2^ = 78%), respectively. Among multi-drug resistant organisms, methicillin-resistant *Staphylococcus aureus,* carbapenem-resistant *Acinetobacter baumannii, Klebsiella pneumoniae, Pseudomonas aeruginosa* and multi-drug resistant *Candida auris* were most commonly reported. Stratified analyses found higher proportions of AMR outside of Europe and in ICU settings, though these results were not statistically significant. Patient-level analysis demonstrated > 50% (n = 58) mortality, whereby all but 6 patients were infected with a resistant organism.

**Conclusions:**

During the first 18 months of the pandemic, AMR prevalence was high in COVID-19 patients and varied by hospital and geography although there was substantial heterogeneity. Given the variation in patient populations within these studies, clinical settings, practice patterns, and definitions of AMR, further research is warranted to quantify AMR in COVID-19 patients to improve surveillance programs, infection prevention and control practices and antimicrobial stewardship programs globally.

**Supplementary Information:**

The online version contains supplementary material available at 10.1186/s13756-022-01085-z.

## Background

The pandemic caused by severe acute respiratory syndrome coronavirus 2 (SARS-CoV-2) virus has been one of the most significant challenges of our time and has overwhelmed healthcare systems worldwide [1]. Simultaneously, the rise in multi-drug resistant infections continues to threaten global heath through significant morbidity, mortality and global economic loss. Following the O’Neill review and recommendations in 2016 to respond to the antimicrobial resistance (AMR) crisis [1], important progress has been made. However, patient admissions to hospitals have contributed to and continue to increase the risk of health-care-associated infections and the transmission of multidrug-resistant (MDR) organisms. Recent evidence suggests that as a consequence of the coronavirus disease 2019 (COVID-19) pandemic [2], an increasing number of patients admitted to hospitals have been prescribed empirical antimicrobial therapy which may not always be appropriate [3–6], potentially increasing the number of resistant infections globally.

While treatment of COVID-19 with antimicrobials is ineffective, there are several reasons why antimicrobial prescribing may exist [7, 8]: patients may present with symptoms similar to that of bacterial or other viral pneumonias, there may be suspected or confirmed co-infections [4], and protocols and existing healthcare frameworks might suggest the use of antimicrobials [9]. While antimicrobial therapy in COVID-19 patients may be reasonable if bacterial or fungal infection is suspected, consideration for AMR and antimicrobial stewardship focused on supporting the selection of optimal empirical therapies and appropriate de-escalation or discontinuation of antimicrobials when bacterial co-infection is present or absent is important [7].

A growing body of evidence suggests AMR may be increasing following antimicrobial prescribing in COVID-19 patients^10–13^, but a quantification of the prevalence of AMR and relative proportions of associated pathogens within a systematic review has not been published to date. Understanding the emergence of AMR in COVID-19 patients is essential. There is clear evidence to suggest that excess antimicrobial use in humans leads to antimicrobial resistant microbes that negatively impact humans, and AMR is described as one of the top ten greatest threats to global public health, food security, and development [11]. An important knowledge gap exists regarding the prevalence, and characteristics of bacterial and fungal co-infections, including potential AMR in patients with COVID-19.

We conducted a systematic review and meta-analysis of the published literature to address the specific research question: “*What is the prevalence of AMR in co-infected COVID-19 patients?*” Our objective was to identify and characterize the available literature by (1) reviewing COVID-19 patients with co-infections, (2) assessing the healthcare settings and geography (3) documenting antimicrobial therapies prescribed including antibacterials and antifungals if available, and (4) estimating the proportion of resistant organisms reported in the literature.

## Methods

### Search strategy and selection criteria

We conducted a systematic review following the Preferred Reporting Items for Systematic Reviews and Meta-analyses (PRISMA) guidelines for reporting [see Additional file 1] [13]. The study protocol was registered with the PROSPERO database for systematic reviews: CRD42021227564.

Searches of MEDLINE, Embase, Web of Science (BioSIS), and Scopus were completed for literature published from November 1, 2019 to May 28, 2021. These searches were restricted to human studies and publications in the English language. A separate search of medRxiv for unpublished manuscripts was also conducted to ensure the search was comprehensive. The search strategy was designed to capture original research articles with a focus on human studies involving confirmed COVID-19 patients with co-infections that reported drug-resistant organisms. Reference lists from all articles included in the review were reviewed to identify additional studies. The complete search strategy is presented in Additional file 2.

Results from each database search were uploaded into Covidence [14], an online software platform for systematic reviews, which removed all duplicate articles. Title and abstract screening were divided among three authors (RMK, DAJ, DCJ) and conducted independently to identify potential articles for inclusion, with each article requiring approval from at least two authors prior to moving to full-text review. Eligibility conflicts were resolved by a fourth author (SLL) who was not involved in the initial screening. Manuscripts were excluded if a duplicate was missed by Covidence, lack of resistant organisms were reported, publication in a language other than English, evaluation in non-COVID-19 patient populations, inappropriate study design (as listed below), inappropriate comparators or inappropriate outcomes such as antibiotic prescribing. The same process was applied for full-text screening. Studies, including case reports, cohort studies, case series, case–control studies, and conference proceedings, were included if antimicrobial resistant organisms were documented among human patients with confirmed COVID-19 requiring hospital care, accompanied by either a laboratory confirmed co-infection, or an existing co-organism isolated from a site thought to be associated with infection as stated by the study authors. Editorials, commentaries, in vitro studies (non-clinical isolates, animal studies, mechanistic studies), reviews, and studies in which none of the patients had COVID-19 were excluded.

Data extraction was performed by the same three authors involved in study selection, and information was recorded relating to study details (author, geographic location, study design, sample size), demographics (healthcare setting, age, sex), clinical parameters (disease presentation, mechanical ventilation, comorbidities, antimicrobials used; type, length) and microbiology (organisms identified, method of identification, antimicrobial susceptibility testing method, antimicrobial resistance, definition of resistance), as available. Patient-level data were also collected if provided. Upon independent completion of the initial data extraction, articles were again divided among the three authors (RMK, DAJ, DCJ) and reviewed a second time to ensure accuracy and comprehensiveness of the extraction.

Given the lack of a standardized definition for co-infection or secondary infection, superinfection or colonization across studies, the authors’ reporting of any type of co-infection or secondary infection was used. For purposes of our study co-infection was defined as simultaneous infection with a virus, bacterial or fungal organism in addition to SARS-CoV-2 either at the time of presentation or during the course of hospitalization. Similarly, given the lack of standardized definitions for AMR across all articles, we defined antimicrobial resistance as per authors’ discretion whereby microbiological investigations provided evidence of resistance, and whether or not specific guidelines or interpretative criteria such as Clinical & Laboratory Standards Institute (CLSI) or European Committee on Antimicrobial Susceptibility Testing (EUCAST) were used.

### Data analysis

The studies included in the review were assessed for risk of bias using the Joanna Briggs Institute (JBI) Critical Appraisal Tools (specifically the case report, case series, cross-sectional, and cohort study checklists) (see Additional file 3) [15]. Select questions from the QUADAS-2 Tool [16], specifically, Domain 3: reference standard, were also used to assess the diagnostic methods for organism identification across all studies (see Additional file 4). These assessments were performed by three authors (RMK, DAJ, DCJ), with each article assessed by two authors for completion. Issues encountered while conducting the assessments due to unspecified study designs were resolved by discussion among the authors conducting the assessments, and the most suitable checklist was chosen for these studies.

Descriptive statistics including means and ranges were reported for continuous outcomes. Dichotomous outcomes were reported as frequencies and proportions calculated on GraphPad Prism v8.2 (La Jolla, California, USA). Study-level analysis was reported either narratively (number of co-infecting organisms, number of co-infections, number of resistant co-infections, microbiological identification and resistance reporting methods) or with a formal meta-analysis where appropriate. Using the ‘metaprop’ command within Stata 16 statistical software (College Station, TX: StataCorp LLC), we calculated pooled prevalence estimates (with corresponding 95% confidence intervals (CIs)) of co-infection with resistant bacterial and fungal organisms separately using random effects models. We visualized all pooled estimates with forest plots and assessed between-study heterogeneity using the *I*^2^ statistic, which indicates the percentage of variation among the studies that occurs as a result of heterogeneity rather than chance. Variation across all studies was categorised as low (*I*^2^ < 25%), moderate (*I*^2^ between 50 and 75%), high (*I*^2^ > 75%), or no statistical heterogeneity (*I*^2^ = 0%). Given the variation in geographic and hospital settings among included studies, we also conducted stratified meta-analysis by the following variables: ICU vs. non-ICU settings, COVID-specific ICU vs. regular ICU or other hospital setting, North America vs. Europe vs. Other, Europe vs. Asia vs. Other and Italy vs. Europe (excluding Italy) vs. Other.

Patient-level analysis was reported narratively (age, sex, number of co-infecting organisms, number of co-infections, number of resistant co-infections, proportion of patients receiving antibiotic therapy, outcome) using descriptive statistics. Case reports, studies evaluating effectiveness of COVID-19 therapies such as tocilizumab, and those reporting MDR rates where patient-level data could not be interpreted were excluded (n = 12) for the narrative reporting.

### Role of the funding source

The Antimicrobial Resistance—One Health Consortium is funded through the Major Innovation Fund Program of the Ministry of Jobs, Economy, and Innovation (JEI), Government of Alberta, Canada.

## Results

**Fig. 1:**
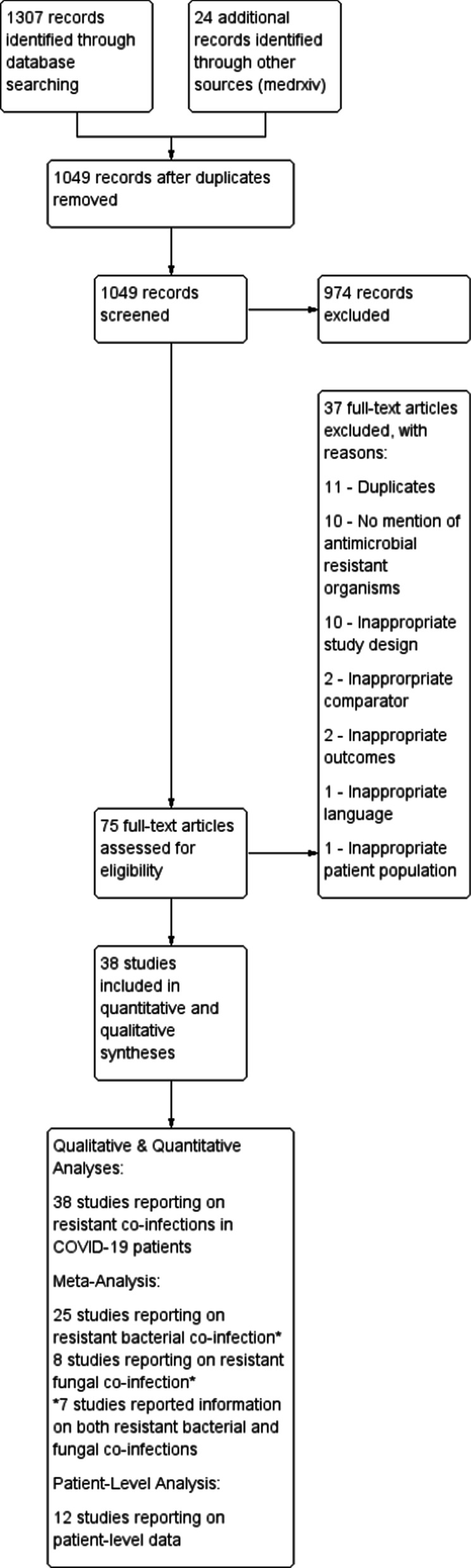
PRISMA flow diagram

The search strategy identified 1331 records from MEDLINE (n = 330), Embase (n = 296), Web of Science (n = 96), Scopus (n = 585), and an additional 24 records through medRxiv (Fig. 1). After removal of duplicates, 1049 articles remained for title and abstract screening. Seventy-five articles were eligible for full-text screening of which 38 met inclusion criteria (Fig. 1). Thirty-seven articles were excluded due to missed duplicates (missed by Covidence) during the initial automated de-duplication process (n = 11), lack of resistant organisms reported (n = 10), inappropriate study design as listed in the Methods (n = 10), inappropriate comparator (n = 2), inappropriate outcomes such as antibiotic prescribing (n = 2), wrong language (n = 1) and non-COVID-19 patients (n = 1). Geographical origin of the 38 studies (Table 1) was as follows: Belgium (n = 2), China (n = 1), Egypt (n = 1), France (n = 3), Greece (n = 1), India (n = 2), Italy (n = 11), Iran (n = 3), Mexico (n = 1), Saudi Arabia (n = 1), Spain (n = 4), Switzerland (n = 1), Qatar (n = 1), United Kingdom (n = 2), and United States (n = 4). Twenty-seven (71%) studies enrolled patients from the intensive care unit (ICU), whereas 6 (16%) studies enrolled patients from COVID-specific care units, and 5 (13%) studies had an unspecified setting. The following study designs were identified: retrospective cohort (n = 8), case series (n = 5), case report (n = 3), cross-sectional (n = 3), prospective observational (n = 1), prospective cohort (n = 3), retrospective observational (n = 12), and 3 case–control studies. Sample sizes ranged from 1 to 4267 patients. Twelve studies contained patient-level data for 112 individuals (Table 1). Patient characteristics in studies meeting inclusion criteria are presented in Table 1. Risk of bias (Additional file 3) revealed the overall quality of the included studies. Majority of the studies were poor with limited reporting of microbiological detail including sample type, microbiological investigations, antimicrobial susceptibility testing methods, and definitions of resistance.

**Table 1:** Summary of study characteristics of the 38 included studies

First Author	Location	Hospital setting	Study type	Microbiological detection and/or identification	Antimicrobial susceptibility testing method
Chowdhary [31]	New Delhi, India	ICU	Retro-spective Cohort	MALDI-ToF MS	CLSI Broth Microdilution Method M27-A3/S4
Bogossian [44]	Brussels, Belgium	ICU	Retro-spective Case Control	Routine surveillance swabs: chromID® CARBA SMART agar, MacConkey agar containing ceftazidime chromID® VRE agar, MALDI-ToF MS	EUCAST: VITEK 2 and disk diffusion Carbapenemases OXA-48, KPC, NDM, VIM and IMP; VanA, VanB were detected via PCR analysis or Coris Resist-5 O.O.K.N.V. antigenic. MDR *Pseudomonas* spp. and *Acinetobacter* spp. were defined as recommended considering antimicrobial resistance phenotype
Ramadan [33]	Assiut, Egypt	Tertiary hospitals: Alrahji Liver Hospital and Assiut University Hospital	Prospective Cohort	MALDI-ToF MS	Detection of antibiotic resistance genes by Monoplex PCR Technique (*mecA, NDM-1, KPC, TEM, CTX-M, SHV)*
Amarsy [45]	Nantes, France	ICU	Retro-spective Cohort	Blood cultures and respiratory cultures	Detection of resistance genes by Illumina WGS
Perez [19]	New Jersey, USA	ICU, Medical-surgical unit, progressive care unit	Retro-spective Cohort	Clinical specimens, colonization screening	CRAB definition: Detection using RT-PCR for carbapenemase genes
Salehi [22]	Tehran, Iran	Three tertiary care training hospitals	Cross-Sectional	Budding yeasts and pseudohyphae in KOH 10% preparation and culture	CLSI M60 and M59 supplements
Cataldo [46]	Rome, Italy	ICU	Retro-spective Cohort	Blood cultures	NA
Posteraro [47]	Basel, Switzerland	COVID care unit	Case Report	MALDI-ToF MS	Sensititre YeastOne® method confirmed by the CLSI M27-A3 reference method
Nori [3]	New York, City, NY, USA	ICU	Retro-spective Observa-tional	Respiratory cultures, blood cultures	NA
Mahmoudi [48]	Hamedan, Iran	Nahavand Hospitals	Cross-Sectional	Blood and endotracheal aspirate samples	CLSI
Li [22]	Wuhan, China	Hospital (designated for COVID patients)	Retro-spective Cross-Sectional	Qualified sputum, endotracheal aspirate, bronchoalveolar lavage fluid, blood samples, or qualified urine	CLSI
Contou [18]	Argenteuil, France	COVID ICU	Retro-spective Cross-Sectional	Blood cultures, cultures of the respiratory tract secretions	Panel RP2 plus (Film Array Biomerieux®), Panel Pneumonia Plus (Film Array Biomerieux®)
Mo [22]	Brooklyn, New York	Community Teaching Hospital	Case Series (Retro-spective Observa-tional)	NA	NA
Garcia-Menino [8]	Oviedo, Spain	ICU	Case Series (Retro-spective Observa-tional)	MALDI TOF/MS -	Microscan System (BeckmanCoulter, Brea, CA, USA); results interpreted according to EUCAST
Sharifipour [20]	Qom, Iran,	ICU	Prospective Cohort	Samples were cultured on Blood Agar, Chocolate Agar, Eosin Methylene Blue (EMB), and MacConkey Agar	CLSI
Walpole [50]	United Kingdom	ICU	Case Report	Sputum sample	NA
Razazi [10]	France	ICU	Retro-spective Cohort	Bacterial co-infection at ICU admission evidenced by detection of bacteria in sputum or blood samples, in the absence of other sources of infection, or by a positive pneumococcal or *L. pneumophila* serotype 1 urinary antigen test	Susceptibility profiles of recovered microorganisms were recorded
Guisado-Gil [17]	Seville, Spain	ICU	Retro-spective Cohort	Blood cultures obtained > 48 h after admission	EUCAST. MDR categorization according to the Germany Society for Hygiene and Microbiology
Montrucchio [51]	Turin, Italy	ICU	Case Series (Retro-spective Observa-tional)	MALDI-ToF MS	EUCAST: Microscan WalkAway plus System, MASTDISCS® *Combi Carba plus* disk system
Mady [23]	Riyadh, Saudi Arabia	ICU	Case Series (Retro-spective Observa-tional)	Blood and respiratory cultures	Not described
Tiri [52]	Terni, Italy	ICU	Retro-spective Observa-tional Cohort	MALDI-ToF MS	VITEK2; Immunochromatography for OXA-48-like, OXA-163, KPC, NDM, VIM
Kokkoris [53]	Athens, Greece	ICU	Case Series (Retro-spective Observatio-nal Cohort)	Blood specimen	NA
Perrotta [54]	FG, Italy	ICU	Case Report	NA	NA
Baiou [55]	Doha, Qatar	ICU	Retro-spective Case–Control	MALDI-ToF MS	BD Phoenix according to CLSI standards
Segrelles-Calvo [56]	Madrid, Spain	ICU, RICU	Prospective Observa-tional Cohort	*Aspergillus* galactomannan antigen on BAL	Not described
Martinez-Guerra [57]	Mexico City, Mexico	COVID-19 dedicated facility	Prospective Cohort	MALDI-Tof MS	VITEK2; AmpC producers considered with known chromosomal AmpC Beta-Lactamases, ESBL considered in those resistant to 3^rd^ generation cephalosporins and monobactams, CRE considered with resistance to carbapenems in VITEK and confirmed with modified CIM test, MDR *P. aeruginosa* considered in isolates with resistance to at least one agent in three or more antibiotic categories
Karruli [58]	Naples, Italy	ICU	Retro-spective Observa-tional Cohort	Microbiological sampling of blood, urine, and airways	MDR defined according to Magiorakos et al. [59] criteria
Gomez-Simmonds [60]	New York City, USA	ICU	Retro-spective Observational Cohort	Surveillance using MicroScan	Xpert Carba-R, BMD, E-test, WGS
Cultrera [61]	Ferrara, Italy	ICU	Retro-spective Observational Cohort	MALDI-ToF MS	VITEK2
Khurana [62]	New Delhi, India	COVID-19 dedicated facility	Retro-spective Observational Cohort	VITEK2 and BioFire FilmArray Respiratory Panel	VITEK2 AST card interpreted by CLSI guidelines
Posteraro [47]	Rome, Italy	ICU	Retro-spective Observa-tional Cohort	Positive blood culture using BacT/ALERT VIRTUO and MALDI Biotyper	VITEK2 and Sensititre YeastOne® following EUCAST breakpoints
Pascale [63]	Bologna, Italy	ICU and non-ICU settings	Cross-Sectional	Active surveillance of blood and respiratory cultures	CRE defined as per EUCAST criteria; WGS
Baskaran [64]	England	ICU	Multicentre Retro–spective Observa-tional Cohort	Standard culture, respiratory viral PCR and urinary antigen tests	NA
Moretti [65]	Brussels, Belgium	ICU	Retro-spective Observational Cohort	Endotracheal aspiration or BAL with > 10^5^ and > 10^4^ CFU/mL	Not described. MDR or extreme-drug resistant (XDR) based on European Center of Disease Prevention and Control (ECDC)
Grasselli [66]	Genoa, Italy	ICU	Retro-spective Observa-tional Cohort	Routine microbiological surveillance: perineal swabs, nasal swabs, tracheal aspirate, urine culture	Not described
Magnasco [67]	Genoa, Italy	ICU	Retro-spective Observa-tional Cross-Sectional	Blood, respiratory and urinary samples using VITEK MS	VITEK2; Sensititre YeastOne® Panel (antifungal)
Bentivegna [68]	Rome, Italy	COVID-19 Depart-ment	Case–Control	NA	NA
Suarez-de-la-Rica [69]	Madrid, Spain	CCU	Retro-spective Observational Cohort	Conventional culture	Not described

Abbreviations: Bronchoalveolar Lavage (BAL), Clinical and Laboratory Standards Institute (CLSI), Critical Care Unit (CCU), European Committee on Antimicrobial Susceptibility Testing (EUCAST), intensive care unit (ICU), multi-drug resistant (MDR), matrix-assisted laser desorption ionization-time of flight mass spectrometry (MALDI-TOF MS), polymerase chain reaction (PCR), whole-genome sequencing (WGS), carbapenem resistant *Acinetobacter baumannii* (CRAB), respiratory intensive care unit (RICU), real-time polymerase chain reaction (RT-PCR), oropharyngeal candidiasis (OPC)

Twelve (32%) articles documented the use of matrix-associated laser desorption ionization time of flight mass spectrometry (MALDI-ToF MS) as a method of organism identification, whereas 26 (68%) articles did not report microbiological investigations beyond specimen type (e.g., blood culture, respiratory culture etc.) and basic culturing (blood agar plate, chocolate agar plate etc.). Three studies had no description of microbiological investigations whatsoever (Table 1). Antimicrobial resistance definitions varied across studies, with the majority of articles not explicitly defining resistance, hence the authors’ final interpretation of resistance per isolate was used. One study reported multi-drug resistant categorization according to the Germany Society for Hygiene and Microbiology [17]. Details of antimicrobial susceptibility testing varied across studies, with 13 (34%) articles documenting use of standardized protocols. Seven articles followed CLSI criteria whereas 6 articles followed the EUCAST interpretive criteria. Moreover, reporting of resistance mechanisms were poor, with many reporting both acquired and intrinsic resistance as resistance (Table 2).

**Table 2:** Proportion of COVID-19 patients with resistant co-infections

First author	Disease presentation	Patients screened (No.)	SARS-CoV-2 patients (No.)	Number of patients with co-infections (%)^*^	Number of patients with resistant co-infections (%)^*^
Chowdhary [31]	BSI	596	596	15 (2.5%)	10 (1.7%)
Bogossian [44]	COVID-19	75	69	24 (34%)	23 (33%)
Ramadan [33]	COVID-19	260	260	28 (11%)	28 (11%)
Amarsy [45]	BSI, Respiratory Distress	96	95	4 (45%)	4 (4%)
Perez [19]	VAP, VAP with bacteremia, bacteremia, bone or soft tissue infections	34	17	17 (100%)	17 (100%)
Salehi [22]	OPC	1059	1059	53 (5.0%)	2 (0.2%)
Cataldo [46]	BSI	57	57	28 (49%)	9 (16%)
Posteraro [47]	79 M with complicated T2DM who had fever, necrotic and ulcerative lesions on the amputated leg stump, BSI	1	1	1 (100%)	1 (100%)
Nori [3]	COVID-19 and subsequent positive microbiological results (within 30 days)	4267	4267	152 (3.6%)	25 (0.6%) †
Mahmoudi [48]	COVID-19 patients assessed for bacterial infections	340	340	43 (13%)	31 (72%)
Li [49]	Lung, BSI and UTI	1495	1495	102 (6.8%)	102 (6.8%)‡
Contou [18]	All microbiological investigations performed within the first 48 h of ICU admission were reviewed	92	92	26 (28%)	7 (8%)§
Mo [22]	COVID-19 patients who received Tocilizumab	617	38	15 (39%)	11 (29%)
Garcia-Menino [8]	Suspected or confirmed COVID-19 Patients	62	62	7 (11%)	7 (11%)
Sharifipour [20]	COVID-19 Patients	19	19	19 (100%)	17 (89%)
Walpole [50]	33 M with fever for 3 days, abdominal pain for 1 day and one episode of vomiting	1	1	1 (100%)	1 (100%)
Razazi [10]	Viral ARDS	3821	90	NA	21 (23%)
Guisado-Gil [17]	Hospital-acquired Candidemia and MDR BSI	282	282	NA¶	NA¶
Montrucchio [51]	COVID patients screened for carbapenemase-producing *K. pneumoniae*	35	7	6 (86%)	6 (86%)
Mady [23]	COVID-19 patients with ARDS receiving tocilizumab	61	61	12 (20%)	3 (5%)
Tiri [52]	Patients admitted to ICU screened using rectal swabs or clinical cultures for CRE	62	62	17 (27%)	17 (27%)
Perrotta [54]	57 M admitted to hematology with *K. pneumoniae* NDM sepsis	1	1	1 (100%)	1 (100%)
Baiou [55]	Critical COVID-19 Patients	1231	234	78 (6%)	NA
Segrelles-Calvo [56]	Adult patients admitted to ICU or RICU	215	215	7 (3%)	NA
Martinez-Guerra [57]	Severe COVID-19 patients	794	794	74 (11%)	127 (20%)††
Karruli [58]	Critically Ill COVID-19 Patients	32	32	NA/32	16 (50%)
Gomez-Simmonds [60]	Secondary CPE infections in COVID-19 patients	3152	3152	NA	13 (0.4%)
Cultrera [61]	COVID-19 patients admitted in ICU and non-COVID-19 ICU settings	NA	NA	28	NA
Khurana [62]	Severely ill COVID-19 patients	1179	1179	151 (13%)	105 (9%)
Posteraro [70]	BSI in COVID-19 patients	293	293	46 (16%)	12 (5%)
Pascale [63]	> 18 years admitted to ICU	1151	1151	NA/1151	23 (1.8%)
Baskaran [64]	COVID-19 patients in ICU	579	254	83 (33%)	NA/254
Moretti [65]	Patients with COVID-19 and VAP	39	39	21 (54%)	67%#
Grasselli [66]	Patients with COVID-19 pneumonia	813	813	359 (44%)	38%**
Magnasco [67]	Patients with severe COVID-19	118	118	NA/118	14 (12%)
Bentivegna [68]	Patients in COVID-19 Departments	NA	NA	NA/NA	150/NA
Suarez-de-la-Rica [69]	Mechanically ventilated critically ill COVID-19 patients	107	107	46 (43%)	17 (16%)
Kokkoris [42]	BSI in COVID-19 patients	50	50	27 (54%)	17 (34%)‡‡
**TOTAL**		23,086	16,602		

Abbreviations: BSI (blood stream infection), VAP (ventilator-associated pneumonia), OPC (oropharyngeal candidiasis), MDR (multi-drug resistant), T2DM (type II diabetes mellitus), UTI (urinary tract infection), ARDS (acute respiratory distress syndrome), CRE (carbapenem-resistant *Enterobacteriaceae)*, RICU (Respiratory Intermediate Care Unit)

^*^Denominator: Patients with SARS-CoV-2

^†^24 organisms resistant; no patient level data provided

^‡^159 organisms detected; no patient level data provided; however, all resistant

^§^7 organisms resistant to 3^rd^ generation cephalosporins and amoxicillin-clavulanate, no patient level data

^¶^Rates of MDR BSIs

^#^67% of 27 organisms isolated were MDR, 1 was XDR

^**^272/723 microbiologically confirmed hospital acquired infections were MDR

^††^19.3% (127/656) episodes of hospital-acquired infections demonstrated resistance

^‡‡^34% (17/50) were reported as extensively drug-resistant, pan-drug resistant or resistant

In total, 16,602 (72%) of 23,086 patients had laboratory-confirmed SARS-CoV-2 infection. The proportion of co-infection with either bacterial or fungal organisms in those with confirmed SARS-CoV-2 ranged from 2.5 to 100% across the 35 studies with exclusion of the single case reports. (Table 2). There were no reports of parasitic co-infections. Moreover, 1 case of viral co-infection was captured by our search strategy. One study evaluated viral co-infections using the Cepheid Xpert Xpress Flu/RSV, Panel Pneumonia Plus Film away and Panel RP2 plus Film array and found no cases of viral co-infection in 68 patients, whereas another detected metapneumovirus using the Biofire Film Array [18]. Two cohort studies [19, 20] reported co-infection prevalence of 100%. In contrast, 8 studies [3, 10, 21, 22] (with sample sizes greater than 1000) reported prevalence of co-infection from 3.6 to 13%.

The range of those co-infected with a resistant organism was 0.2 to 100%, with the 4 cohort studies [19, 20] contributing to the higher limit as previously mentioned (Table 2). Studies with larger sample sizes (> 1000) had resistant co-infection estimates ranging from 0.2 to 9%. In 15 studies where blood stream infections, acute respiratory distress syndrome or ventilator-associated pneumonia was reported, total resistant co-infections ranged from 1.7 to 100%. Seven studies reported both bacterial and fungal infections in COVID-19 patients. Notably, one study evaluated 61 patients who received tocilizumab, of whom 3 (5.0%) had resistant bacterial co-infections [23].

**Fig. 2:**
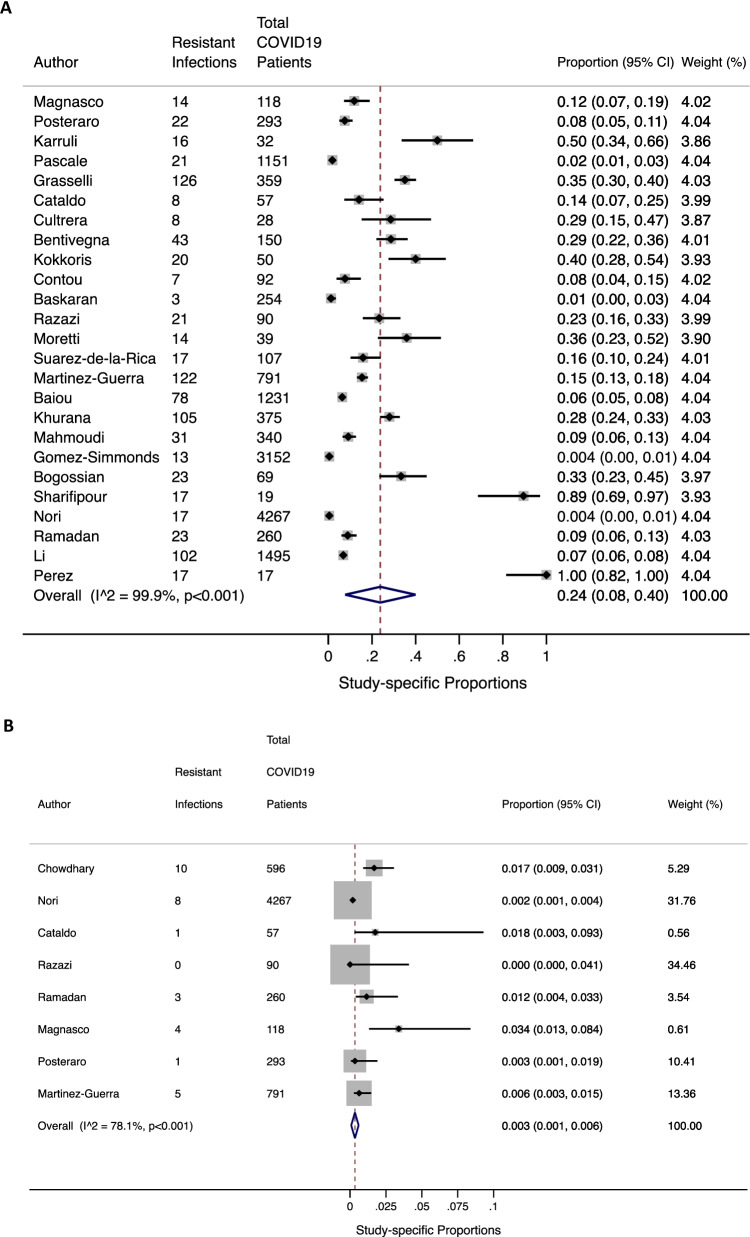
**A** Studies reporting resistant bacterial infections (n = 25); **B** studies reporting resistant fungal infections (n =85)

**Fig. 3:**
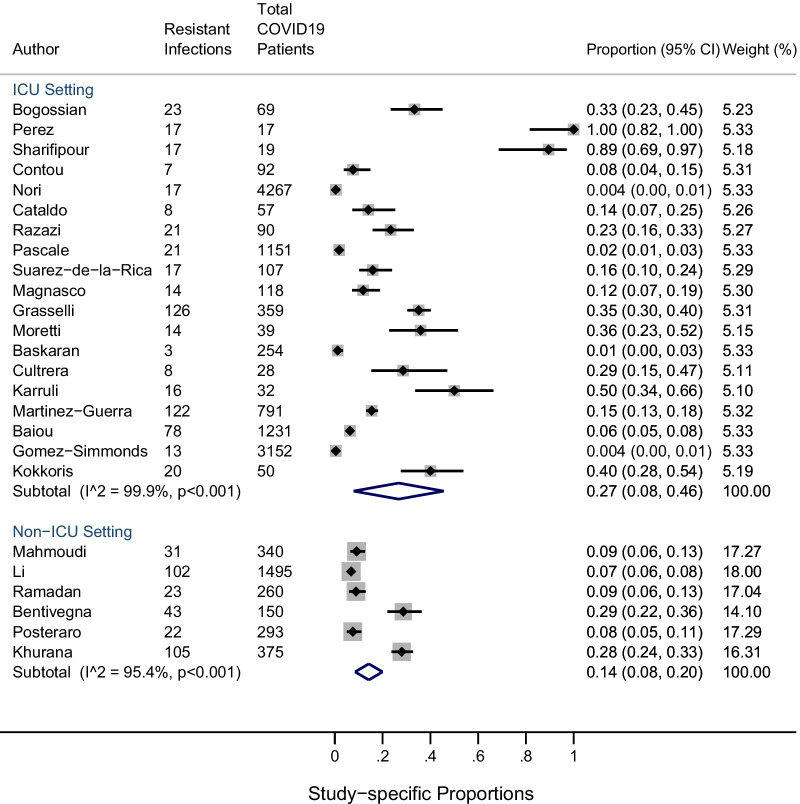
Proportion of resistant infections among ICU and non-ICU settings

**Fig. 4:**
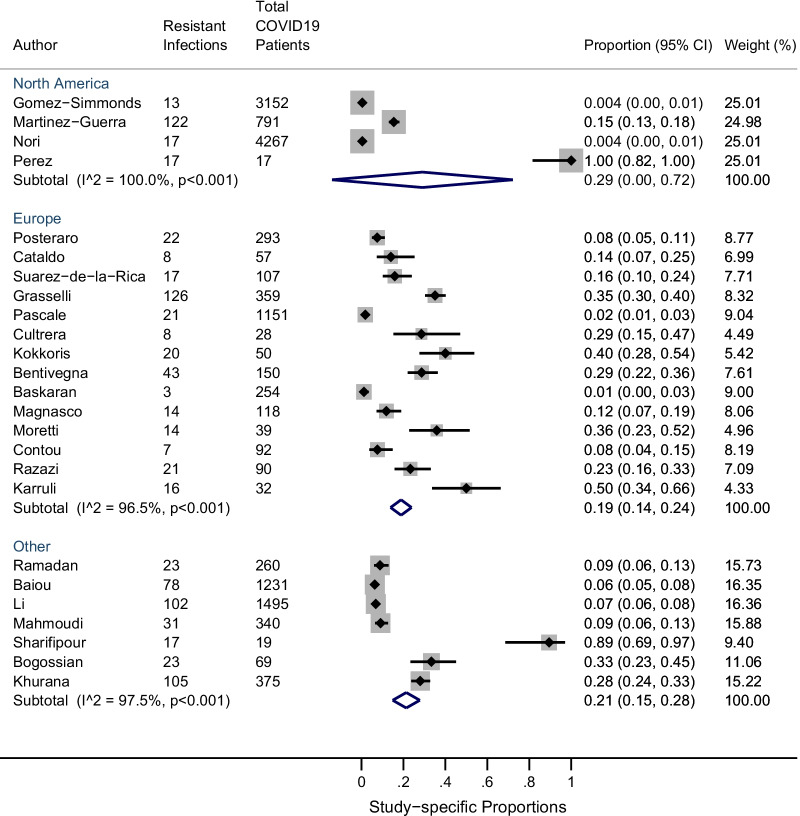
Proportion of resistant infections among North America, Europe and other geographical settings

The pooled prevalence of co-infection with resistant bacterial and fungal organisms was 24% (95% CI 8–40%; n = 25 studies: I^2^ = 99%) and 0.3% (95% CI 0.1–0.6%; n = 8 studies: I^2^ = 78%) respectively (Fig. 2sA and B) . Between-study heterogeneity across bacterial and fungal resistant co-infections was high. Stratified meta-analysis by ICU setting among resistant bacterial infections revealed that the overall proportion of resistant infections amongst COVID19 patients was higher in the ICU setting (n = 19) [0.27 (95% CI 0.08, 0.46)] compared to the non-ICU settings (n = 6) [0.14 (95% CI 0.08, 0.20)], although not significant (Fig. 3). Furthermore, comparison between regular ICU or hospital settings (n = 22) to COVID-specific ICUs (n = 3) showed a similar trend ([0.25 (95% CI 0.07, 0.42)] vs. 0.19 [0.14 (95% CI 0.07, 0.22)]) (see Additional file 5). Moreover, a stratified analysis was performed by geography whereby the prevalence of resistant bacterial infections in studies conducted outside Europe [0.19 (95% CI 0.14, 0.24)] was higher, particularly in Asia [0.21 (95% CI 0.15, 0.28)] and more prominent in North America [0.29 (95% CI: 0.00, 0.72)] although not significant (Fig. 4, Additional files 6–7). Again, statistical heterogeneity remained high across all stratified analyses.

There were 1959 unique organisms identified across 387 studies where data were available, with 569 (29%) organisms identified as resistant to one or more antimicrobials (Table 3). The most common Gram-negative organisms resistant to at least one antimicrobial (regardless of intrinsic resistance) were *Klebsiella pneumoniae* (n = 169), *Acinetobacter baumannii* (n = 148), *Pseudomonas aeruginosa* (n = 65), *Escherichia coli* (n = 43)*, Enterobacter cloacae* (n = 29)*, Stenotrophomonas maltophilia* (n = 24) and *Serratia marcescens* (n = 17). Wide-spread resistance mechanisms were documented including β-lactamases, carbapenemases, and extended spectrum β-lactamases (ESBLs). The most common resistant Gram-positive organisms included: methicillin-resistant *Staphylococcus aureus* (MRSA) (n = 132), coagulase-negative staphylococci (n = 30) and vancomycin-resistant *Enterococcus* (VRE) spp. (n = 10). More specifically, isolates of *E. faecium* were documented with high levels of vancomycin resistance. Resistance to at least one antifungal agent was also documented, including *Candida auris* (n = 10), *C. albicans* (n = 3) and unidentified *Candida* spp. (n = 5).

**Table 3:** Co-infecting organisms and resistance profiles

Organism	Number	Proportion Resistant	Resistance Phenotype
**Gram-positives**			
*Staphylococcus aureus*	2	0 (0%)	–
Methicillin-sensitive *Staphylococcus aureus* (MSSA)	104	0 (0%)	–
Methicillin-resistant *Staphylococcus aureus* (MRSA)	132	132 (100%)	Methicillin resistance
Coagulase-negative staphylococci (CNS)	99	30	Unknown resistance (24)
Vancomycin-resistant enterococci (VRE), unspecified	4	4 (100%)	Vancomycin resistance
*Enterococcus faecalis*	29	1 (3%)	High-level aminoglycoside resistance
*Enterococcus faecium*	33	6 (18%)	Vancomycin resistance (3); high-level aminoglycoside resistance (3), ampicillin resistance (1)
*Enterococcus casseliflavus/Enterococcus gallinarum*	2	1 (50%)*	Vancomycin resistance (1 – E. gallinarum)*
*Enterococcus* spp.	12	0 (0%)	–
*Streptococcus pneumoniae*	15	3 (20%)	Amoxicillin, amoxicillin/clavulanic acid, cefoxitin, gentamicin, erythromycin, clindamycin, piperacillin/tazobactam, trimethoprim/sulfamethoxazole (2); amikacin, ciprofloxacin, levofloxacin, cefotaxime, ceftriaxone, ceftazidime, cefepime (1), unknown resistance (1)
*Streptococcus* spp.	4	0 (0%)	–
*Clostridium difficile*	7	0 (0%)	–
**Gram-negatives**			
*Acinetobacter baumannii*	218	148 (68%)	Wide-spread resistance except to colistin (98); 26 isolates harbored *OXA*-23, 2 harbored *NDM*; Carbapenem resistance (4); extensively resistant (5), pan-drug resistant (3), unknown resistance (19)
*Klebsiella pneumoniae*	274	169 (62%)	26 (carbapenem-producing *KPC*), *OXA*-48 (7), ESBL (13), *NDM* (1), multi-drug resistant (23), Carbapenem resistance (13), KPC-2 (1), KPC-3 (1), unknown resistance (25), VIM (1)
*Klebsiella oxytoca*	5	2 (40%)	ESBL (2)
*Klebsiella aerogenes*	4	0 (0%)	–
*Klebsiella* spp*.*	9	0 (0%)	–
*Pseudomonas* spp.	4	0 (0%)	–
*Pseudomonas aeruginosa*	203	65 (25%)	Unknown resistance (34), piperacillin/tazobactam (4), carbapenems (18), MDR (6), cephalosporin resistance (1), XDR (1)
*Serratia marcescens*	18	17 (94%)	Resistance to amoxicillin, amoxicillin/-clavulanic acid, 1^st^ and 2^nd^ generation cephalosporins (including AmpC B-lactamase) with low level of resistance to amikacin, multi-drug resistant (7)
*Escherichia coli*	118	43 (36%)	ESBL (2), AmpC resistance (1), multi-drug resistant (12), unknown resistance (18)
*Stenotrophomonas maltophilia*	60	24 (40%)	Multidrug resistant (24)
*Proteus mirabilis*	5	0 (0%)	–
*Proteus putida*	1	0 (0%)	–
*Haemophilus influenzae*	7	0 (0%)	–
*Moraxella catarrhalis*	1	0 (0%)	–
*Enterobacteriaceae,* unspecified	5	0 (0%)	–
*Yersinia enterocolitica*	1	1 (100%)	Amoxicillin and amoxicillin/-clavulanic acid resistance
*Enterobacter* spp.	8	5 (63%)	Imipenem resistance
*Enterobacter aerogenes*	8	8 (100%)	Carbapenem-resistant Enterobacteriaceae (1), ESBL (1), AmpC B lactamase (6), unknown resistance (1)
*Enterobacter cloacae*	68	29 (43%)	AmpC B lactamase (7), multi-drug resistant (18), NDM-1 (2)
*Elizabethkingia meningoseptica*	1	1 (100%)	Multi-drug resistant (1)
*Chryseobacterium gleum*	1	1 (100%)	Multi-drug resistant (1)
*Citrobacter koseri*	1	0 (0%)	–
*Mycoplasma pneumoniae*	2	0 (0%)	–
Enterobacterales	113	34 (30%)	Cephalosporin resistance (2); Carbapenem resistance (3), unknown resistance (29)
*Bacteroides fragilis*	2	0 (0%)	–
**Viral organisms**			
Metapneumovirus	1	0 (0%)	–
**Fungal organisms**			
*Candida auris*	11	10 (91%)	Fluconazole resistance (10), Voriconazole non-susceptible (3); overall 3 multi-azole resistant (fluconazole + voriconazole), 7 multi-drug resistant including 3 to 3 classes of drugs (azoles, amphotericin B and 5-flucytosine) and 4 resistant to 2 classes of drugs (azoles + 5-flucytosine and azoles + amphotericin B)
*Candida albicans*	88	3 (3%)	Fluconazole and voriconazole resistance (3); caspofungin intermediate (2)
*Candida dubliniensis*	6	2 (33%)	Fluconazole resistance (1), caspofungin resistance (1)
*Candida parapsilosis*	25	1 (4%)	Fluconazole resistance
*Candida glabrata*	15	1 (7%)	Pan-echinocandin resistance (1), caspofungin intermediate (7)
*Candida* spp.	22	5 (23%)	Azole resistance (5); Echinocandin resistance (1)
*Pichia kudriavzevii*	1	1 (100%)	Caspofungin and fluconazole resistance
*Aspergillus fumigatus*	3	0 (0%)	–
*Aspergillus flavus*	7	0 (0%)	–
*Aspergillus niger*	2	0 (0%)	–
**Other**	73	20 (27%)	Unknown resistance (9), ESBL (11)

^*^Intrinsic resistance

Clinical data for 112 patients were available from 12 studies, of which sex and age were documented for 72. Fifty-two (72%) patients were male with a median age of 65 years (range 25–86) (see Additional file 8). Sixty-five (80%) had co-morbidities present, with all but 3 patients receiving antimicrobials prior to identification and susceptibility testing of co-infecting organisms for which data were available. Sixteen (45%) patients received tocilizumab, 13 (20%) patients received a combination of steroids and tocilizumab and 37 (56%) received a combination of other drugs. Sixty of 67 (90%) patients were receiving mechanical ventilation with a median duration of 34 days (range 24–46 days). All but 20 patients (colonization) had a co-infecting organism as the cause of their disease presentation in addition to COVID-19. The most commonly identified organisms were: *A. baumannii* (n = 38), *K. pneumoniae* (n = 29), *C. auris* (n = 11), *P. aeruginosa* (n = 7), MRSA (n = 3), *Aspergillus fumigatus* (n = 3), *A. flavus* (n = 2), *A. niger* (n = 2), *E. cloacae* complex (n = 2) and MSSA (n = 2). *S. maltophilia, K. oxytoca, Y. enterocolitica, P. aeruginosa* and *C. glabrata* were documented in 1 patient each. Mixed infections were documented in 5 patients: *P. aeruginosa, C. auris; P. aeruginosa, C. auris,* VRE; *A. baumannii, K. pneumoniae* in 2 patients; MRSA and *C. albicans.* All organisms acquired resistance to at least one antimicrobial except for 10 cases pertaining to (1) MSSA, (2) mixed infection with *A. baumannii* and *K. pneumoniae*, (3), *K. pneumoniae*, (4) *A. fumigatus* (n = 3), (5) *A. flavus* (n = 2), and (6) *A. niger* (n = 2). Overall, mortality was documented in 58 (52%) patients with all but 6 infected with resistant organisms.

## Discussion

In this systematic review and meta-analysis, we analyzed data from over 16,000 patients with microbiologically confirmed COVID-19 admitted to hospitals between November 2019 and June 2021. While the prevalence of co-infections was highly variable based on sampling and setting within each of the included studies, we estimated a pooled prevalence of co-infection with resistant bacterial and fungal organisms of 24% and 0.3% respectively. Further, of the 1959 unique isolates identified within the included studies, 569 (29%) were deemed resistant. Despite the large body of literature describing the potential effects of the COVID-19 pandemic on AMR, this is the first study to summarize data surrounding AMR which may have major implications for current and future antimicrobial stewardship as well highlighting gaps in methods of organism identification and reporting of resistance. The concern for AMR during the first full year of the COVID-19 pandemic appears to be low based on our findings. However, with very few reports and poor-quality data, further research is warranted to better understand the landscape of AMR during COVID-19. Additionally, as the pandemic is still ongoing there will be a need to re-assess these findings as further evidence emerges.

The risk of co-infection in patients with influenza has been well documented, with estimates ranging from 2 to 65% [24]. Our study identified a SARS-CoV-2 co-infection congruent with previously reported systematic reviews and large multi-center studies assessing antimicrobial usage that also captured the prevalence of bacterial co-infection [6, 25]. The true prevalence of AMR is currently lacking in literature, and even prior to influenza or the recent COVID-19 pandemic, it has not been well described. A few studies have documented the prevalence of MRSA co-infections in patients with influenza, ranging from 20 to 48%; however other resistant organisms were not frequently reported [26–28]. Furthermore, reports of co-infections with antimicrobial-resistant Gram-negative organsims during influenza season ranged from 2.2% for carbapenems and up to 21% for fluoroquinolones, not necessarily from patients co-infected with influenza [29]. Moreover, inferences of antimicrobial usage could serve as a strong predictor for AMR [24]. In studies evaluating influenza-associated co-infections, antimicrobial usage ranged from 20 to 50% [6, 25]. During the initial stages of the COVID-19 pandemic, up to 60% of patients were prescribed antimicrobials [6, 25]. At the same time, a number of social distancing and public health measures, coupled with increased public adherence to mandates, reduction in travel and increased hand hygiene may have contributed to a decrease in spread [30]. Although our study did not explicitly capture antimicrobial usage or social and public health measures in place at the time of study, patient-level analysis revealed 95% of patients were prescribed antimicrobials prior or during admission to the hospital. Given the difficulty differentiating viral pneumonia from bacterial pneumonia, it is challenging to avoid unnecessary usage of antimicrobials until confirmation of SARS-CoV-2 is obtained. Given this, it is imperative to quantify true rates of AMR to inform the use of appropriate empiric therapies and to understand the types of resistant co-infections that occur in patients with COVID-19.

A large number of carbapenem-resistant *A. baumannii* (CRAB) and multi-drug resistant *C. auris* was identified from some studies highlighting the urgent need for the development of newer and more robust antimicrobial agents [19, 31]. In addition, large numbers of *Klebsiella pneumoniae* (n = 169), MRSA (n = 132) and MDR *Pseudomonas* spp. (n = 65) infections were noted. A majority of COVID-19 patients received azithromycin– a macrolide with known increasing resistance to both Gram-positive and Gram-negative infections. Globally, macrolides are one of the top 5 antimicrobial classes dispensed by pharmacies, with known increases in resistance. One study has suggested an increase in erythromycin resistance in *S. aureus* (26 vs. 43%), which may be associated with high azithromycin use [32]. However, despite our study not being able to specifically capture resistance to azithromycin, there is the possibility of increases in macrolide resistance as a result the initial empiric therapy used during this pandemic.

A number of studies documented blood stream infections and ventilator-associated pneumonia co-infections; however, it was hard to tease out the differences in co-infecting organisms between these different populations. A few studies have reported respiratory co-infections with *Haemophilus influenzae* and *S. aureus*; however, our analysis only found one case of *H. influenzae* co-infection with very low rates of *Streptococcus pneumoniae* co-infections [33]. Conversely, a large number of *S. aureus* bacteremia and candidemia were reported, the latter of which may have been a result of prolonged antimicrobial usage.

A large number of studies were conducted in ICU settings, which numerically reported higher rates of AMR compared to non-ICU settings. Although driven by two cohort studies, the likelihood of AMR to be detected in much greater proportions in ICU settings is not uncommon. Pre-COVID-19, patients admitted to ICU settings are at an increased risk of acquiring infections, with a number of studies citing nosocomial infections in 20–50% of ICU admissions [34–36]. Given the COVID-19 pandemic, where a priori patients are given a combination of antimicrobial and immunosuppressive agents, it is unsurprising to find higher co-infection rates, and in particular, those of resistant nature, especially in patients who have been mechanically ventilated for long periods of time. Moreover, there are geographical differences that increase the risk of acquiring AMR infections, particularly in areas of low- and middle-income countries, poor clean water and sanitation facilities, high movement of livestock and food products as well as lack of routine surveillance in these areas that contribute to the overall inflation of AMR [37]. Our study also demonstrated slightly higher rates of AMR in settings outside of Europe, particularly Asia and some settings in North America. Surveillance programs, robust testing using standardized protocols and reporting; and importantly multimodal strategies focusing on the stringent use of antibiotics in combination with infection, prevention and control practices could enhance antimicrobial stewardship in certain settings, ultimately reducing mortality and morbidity, especially in patients with COVID-19. Recent studies have suggested the use of these multimodal strategies can be very effective to limit the epidemic spread of resistant microorganisms [38, 39].

Identifying clinical and sociodemographic factors that increase a patients’ risk of developing such co-infections have been established and include: healthcare settings, socioeconomic status, prior antibiotic usage, and length of stay in a hospital setting. A priori identifying patients who are higher risk of developing MDR or XDR infections may improve overall prognosis and outcomes, especially in the context of SARS-CoV-2. Future studies that are prospective in nature with well-designed microbiological investigations would enhance our current understanding of AMR during COVID-19 and what is forthcoming.

Our study has several limitations, the largest being the heterogenous reporting of clinically significant isolates causing co-infection versus secondary infection and clinically insignificant isolates found in colonization and contamination. It is also unclear if identified co-infections were the cause of mortality as opposed to other causes of death such as immune dysregulation and cytokine storm. In addition, given the high risk of bias observed within our included studies, our findings may prove contrary if more rigorous studies with larger sample sizes were available or conducted in the future.

Furthermore, the number of true resistant co-infections is largely underestimated due to asynchronous sampling across studies and sites (or lack of), inappropriate sampling due to administration of antibiotics prior to specimen collection as well as lack of appropriate AST in a number of studies beyond basic culturing [40–42]. Moreover, given the likelihood of COVID-19 upon initial examination, delay in other microbiological investigations in combination with empiric antimicrobial therapy mask the true prevalence of co-infections, let alone resistant co-infections. Taken together, our data, and many others, highlight a small subset of the true burden of co-infections and resistant co-infections, which ultimately impact our understanding of disease progression regarding the timing of therapeutic failure due to resistance [43]. Prospective population-based studies incorporating robust initial and follow-up screening protocols can identify key drivers of resistance in co-infected COVID-19 patients, as well as robust microbiological methods including WGS that may pick up heteroresistance may add to our understanding of the effects in which impact AMR, as well as rates.

More importantly, the varying procedures regarding microbiological identification and antimicrobial susceptibility testing in addition to a lack of standardized AMR definition proved difficult when interpreting results. Studies where standardized procedures, such as CLSI or EUCAST, are applied would make data interpretation much more feasible and allow for potential stratification by patient, geography, or clinical factors in future studies. In our analysis, less than 50% of studies used well-defined interpretive criteria and guidelines such as EUCAST or CLSI. To add, some studies reported highly resistant organisms without resistance profiles, driving the overall rate of AMR down given lack of detail. To add, there is a lack of representation from many low- and middle-income countries (LMICs) and smaller studies which introduce publication and selection bias in our analysis that may differ from articles captured from larger centres across Africa, Asia, Europe and North America. Lastly, meta-analyses were conducted using random effects models in light of known clinical heterogeneity by patient and geographic factors. Thus, the pooled estimates should be interpreted with caution while also highlighting the need for more scientific rigour when it comes to reporting AMR, to truly tease out any differences that may translate to effective clinical and laboratory management, as well as public health policy.

## Conclusions

Overall, microbiologically confirmed AMR during the first 18 months of the COVID-19 pandemic was relatively high among patients with bacterial co-infections. The most common resistance documented was in CRAB, MRSA, *Klebsiella pneumoniae, and Pseudomonas aeruginosa,* although some *C. auris* isolates were also identified. Despite no demonstrative differences across hospital ICU settings and geography, further high-quality research is warranted to truly capture the prevalence of AMR during COVID-19 and beyond.

## Supplementary Information

**Additional file 1**. PRISMA Checklist

**Additional file 2**. Search Strategies

**Additional file 3**. JBI Checklists

**Additional file 4**. QUADAS 2 Checklist

**Additional file 5**. Proportion of resistant infections among regular ICU, or other hospital setting and COVID-specific ICU settings

**Additional file 6**. Proportion of resistant infections among Europe, Asia and other geographical settings

**Additional file 7**. Proportion of resistant infections among Italy, Europe (excluding Italy), and other geographical settings

**Additional file 8**. Patient level data

## Data Availability

All data generated or analysed during this study are included in this published article and its supplementary information files.

## Abbreviations

AMR: Antimicrobial resistance
ARDS: Acute respiratory distress syndrome
BAL: Bronchoalveolar lavage
BSI: Blood stream infection
CCU: Critical care unit
CLSI: Clinical & Laboratory Standards Institute
COVID-19: Coronavirus disease 2019
CRAB: Carbapenem-resistant *Acinetobacter baumannii*
CRE: Carbapenem-resistant *Enterobacteriaceae*
ESBLs: Extended spectrum β-lactamases
EUCAST: European Committee on Antimicrobial Susceptibility Testing
ICU: Intensive care unit
JBI: Joanna Briggs Institute
MALDI-ToF MS: Matrix-associated laser desorption ionization time of flight mass spectrometry
MDR: Multi-drug resistant
MRSA: Methicillin-resistant *Staphylococcus aureus*
OPC: Oropharyngeal candidiasis
PCR: Polymerase chain reaction
PRISMA: Preferred Reporting Items for Systematic Reviews and Meta-analyses
RICU: Respiratory intensive care unit
RT-PCR: Real-time polymerase chain reaction
SARS-CoV-2: Severe acute respiratory syndrome coronavirus 2
T2DM: Type II diabetes mellitus
UTI: Urinary tract infection
VAP: Ventilator-associated pneumonia
VRE: Vancomycin-resistant *Enterococcus* spp
WGS: Whole-genome sequencing
