# Supplementary material for: Antimicrobial resistance (AMR) in COVID-19 patients: a systematic review and meta-analysis (November 2019–June 2021)
Source: Antimicrob Resist Infect Control. 2022 Mar 7;11:45. doi: 10.1186/s13756-022-01085-z (PMC8899460; doi:10.1186/s13756-022-01085-z)
Supplement: Supplementary file 2 — Additional file 2. Search Strategies [file 13756_2022_1085_MOESM2_ESM.pdf]

Medline

# ▲	Searches
1	exp Drug Resistance, Microbial/
2	((microb* or bacteri* or fungus or fungal or virus or viral*) adj3 (resistance or resistant)).mp. [mp=title, abstract, original title, name of substance word, subject heading word, floating sub-heading word, keyword heading word, organism supplementary concept word, protocol supplementary concept word, rare disease supplementary concept word, unique identifier, synonyms]
3	(antimicrobial adj3 resistan*).mp. [mp=title, abstract, original title, name of substance word, subject heading word, floating sub-heading word, keyword heading word, organism supplementary concept word, protocol supplementary concept word, rare disease supplementary concept word, unique identifier, synonyms]
4	1 or 2 or 3
5	((((exp Coronavirus/ or exp Coronavirus Infections/ or (D614G or coronavirus* or corona virus* or ncov* or covid* or sars-cov* or sarscov* or Sars-coronavirus* or Severe Acute Respiratory Syndrome Coronavirus*).mp.) and ((20191* or 202*).dp. or 20190101:20301231.(ep).)) not (SARS or SARS-CoV or MERS or MERS-CoV or Middle East respiratory syndrome or camel* or dromedar* or equine or coronary or coronal or covidence* or covidien or influenza virus or HIV or bovine or calves or TGEV or feline or porcine or BCoV or PED or PEDV or PDCoV or FIPV or FCoV or SADS-CoV or canine or CCov or zoonotic or avian influenza or H1N1 or H5N1 or H5N6 or IBV or murine corona*).mp.) or ((((pneumonia or covid* or coronavirus* or corona virus* or ncov* or 2019-ncov or sars*).mp. or exp pneumonia/) and Wuhan.mp.) or (2019-ncov or ncov19 or ncov-19 or 2019-novel CoV or sars-cov2 or sars-cov-2 or sarscov2 or sarscov-2 or Sars-coronavirus2 or Sars-coronavirus-2 or SARS-like coronavirus* or coronavirus-19 or covid19 or covid-19 or covid 2019 or ((novel or new or nouveau) adj2 (CoV on nCoV or covid or coronavirus* or corona virus or Pandemi*2)) or ((covid or covid19 or covid-19) and pandemic*2) or (coronavirus* and pneumonia)).mp. or COVID-19.rx,px,ox. or severe acute respiratory syndrome coronavirus 2.os.)) and 20191201:20301231.(dt).
6	4 and 5
7	(statistic* or numerical or epidemiol*).mp. [mp=title, abstract, original title, name of substance word, subject heading word, floating sub-heading word, keyword heading word, organism supplementary concept word, protocol supplementary concept word, rare disease supplementary concept word, unique identifier, synonyms]
8	exp Epidemiology/
9	7 or 8
10	4 and 5 and 9

EMBASE

# ▲	Searches
1	(((exp Coronavirus/ or exp Coronavirus Infections/ or (coronavirus* or corona virus* or OC43 or NL63 or 229E or HKU1 or HCoV* or ncov* or covid* or sars-cov* or sarscov* or Sars-coronavirus* or Severe Acute Respiratory Syndrome Coronavirus* or D614G).mp.) not (SARS or SARS-CoV or MERS or MERS-CoV or Middle East respiratory syndrome or camel* or dromedar* or equine or coronary or coronal or covidence* or covidien or influenza virus or HIV or bovine or calves or TGEV or feline or porcine or BCoV or PED or PEDV or PDCoV or FIPV or FCoV or SADS-CoV or canine or CCov or zoonotic or avian influenza or H1N1 or H5N1 or H5N6 or IBV or murine corona*).mp.) or (((pneumonia or covid* or coronavirus* or corona virus* or ncov* or 2019-ncov or sars*).mp. or exp pneumonia/) and Wuhan.mp.) or (coronavirus disease 2019 or 2019-ncov or ncov19 or ncov-19 or 2019-novel CoV or severe acute respiratory syndrome coronavirus 2 or sars-cov2 or sars-cov-2 or sarscov2 or sarscov-2 or Sars-coronavirus2 or Sars-coronavirus-2 or SARS-like coronavirus* or coronavirus-19 or covid19 or covid-19 or covid 2019 or ((novel or new or nouveau) adj2 (CoV or nCoV or covid or coronavirus* or corona virus or Pandemi*2)) or ((covid or covid19 or covid-19) and pandemic*2) or (coronavirus* and pneumonia)).mp. or (coronavirus disease 2019 or severe acute respiratory syndrome coronavirus 2).sh,dj.) and 20191201:20301231.(dc).
2	drug resistance, microbial.mp. or exp antibiotic resistance/
3	(antimicrobial adj3 resistan*).mp. [mp=title, abstract, heading word, drug trade name, original title, device manufacturer, drug manufacturer, device trade name, keyword, floating subheading word, candidate term word]
4	((microb* or bacteri* or fungus or fungal or virus or viral*) adj3 (resistance or resistant)).mp. [mp=title, abstract, heading word, drug trade name, original title, device manufacturer, drug manufacturer, device trade name, keyword, floating subheading word, candidate term word]
5	2 or 3 or 4
6	1 and 5

Scopus

(((TITLE-ABS-KEY ((coronavirus* OR "corona virus*" OR oc43 OR nl63 OR 229e OR hku1 OR hcov* OR ncov* OR covid* OR "sars-cov*" OR sarscov* OR "Sars-coronavirus*" OR "Severe Acute Respiratory Syndrome Coronavirus*" OR d614g))) AND NOT ((TITLE-ABS-KEY ((sars OR sars-cov OR mers OR mers-cov OR "Middle East respiratory syndrome or camel*" OR dromedar* OR equine OR coronary OR coronal OR covidence* OR covidien OR influenza AND virus OR hiv OR bovine OR calves OR tgev OR feline OR porcine OR bcov))) OR (TITLE-ABS-KEY ((ped OR pedv OR pdcov OR fipv OR fcov OR sads-cov OR canine OR ccov OR zoonotic OR "avian influenza" OR h1n1 OR h5n1 OR h5n6 OR ibv OR murine AND corona*))))) OR (TITLE-ABS-KEY ((pneumonia OR covid* OR coronavirus* OR corona AND virus* OR ncov* OR 2019-ncov OR sars*) AND wuhan) OR ((2019-ncov OR ncov19 OR ncov-19 OR 2019-novel AND cov OR sars-cov2 OR sars-cov-2 OR sarscov2 OR sarscov-2 OR sars-coronavirus2 OR sars-coronavirus-2 OR "SARS-like coronavirus*" OR coronavirus-19 OR covid19 OR covid-19 OR "covid 2019" OR ((covid OR covid19 OR covid-19) AND pandemic*2) OR (coronavirus* AND pneumonia)))) OR (TITLE ((novel OR new OR nouveau) AND (cov OR ncov OR covid OR coronavirus* OR corona AND virus OR pandemi*))) OR (ABS ((novel OR new OR nouveau) AND (cov OR ncov OR covid OR coronavirus* OR corona AND virus OR pandemi*))) OR (KEY ((novel OR new OR nouveau) AND (cov OR ncov OR covid OR coronavirus* OR corona AND virus OR pandemi*)))) AND (TITLE-ABS-KEY (drug AND resistance OR (microbial OR antimicrobial AND adj3 AND resistan*) OR ((microb* OR bacteri* OR fungus OR fugal OR virus OR viral*) adj3 AND resistance OR resistant))) AND (LIMIT-TO (PUBYEAR , 2021) OR LIMIT-TO (PUBYEAR , 2019) OR LIMIT-TO (PUBYEAR , 2020))

View less

Web of Science

# 3	136	#2 AND #1<br>*Databases= WOS, BCI, BIOSIS, CABI, CCC, DIIDW, KJD, MEDLINE, RSCI, SCIELO, ZOOREC Timespan=All years*<br>*Search language=Auto*
# 2	161,418	TS=(drug resistance, microbial OR antimicrobial adj3 resistan* OR ((microb* or bacteri* or fungus or fungal or virus or viral*) adj3 (resistance or resistant)))<br>*Databases= WOS, BCI, BIOSIS, CABI, CCC, DIIDW, KJD, MEDLINE, RSCI, SCIELO, ZOOREC Timespan=All years*<br>*Search language=Auto*
# 1	196,034	TS =<br>((Coronavirus or Coronavirus Infections/or (coronavirus* or corona virus* or OC43 or NL63 or 229E or HKU1 or HCoV* or ncov* or covid* or sars-cov* or sarscov* or Sars-coronavirus* or Severe Acute Respiratory Syndrome Coronavirus* or D614G)<br>) not<br>(SARS or SARS-CoV or MERS or MERS-CoV or Middle East respiratory syndrome or camel* or dromedar* or equine or coronary or coronal or covidence* or covidien or influenza virus or HIV or bovine or calves or TGEV or feline or porcine or BCoV or PED or PEDV or PDCoV or FIPV or FCoV or SADS-CoV or canine or CCov or zoonotic or avian influenza or H1N1 or H5N1 or H5N6 or IBV or murine corona*)<br>OR (((pneumonia or covid* or coronavirus* or corona virus* or ncov* or 2019-ncov or sars*) or pneumonia) and Wuhan) or<br>(coronavirus disease 2019 or 2019-ncov or ncov19 or ncov-19 or 2019-novel CoV or severe acute respiratory syndrome coronavirus 2 or sars-cov2 or sars-cov-2 or sarscov2 or sarscov-2 or Sars-coronavirus2 or Sars-coronavirus-2 or SARS-like coronavirus* or coronavirus-19 or covid19 or covid-19 or covid 2019 or ((novel or new or nouveau)<br>adj2 (CoV or nCoV or covid or coronavirus* or corona virus or Pandemi*2)) or ((covid or covid19 or covid-19) and pandemic*2) or<br>(coronavirus* and pneumonia)) or (coronavirus disease 2019 or severe acute respiratory syndrome coronavirus 2))<br>*Databases= WOS, BCI, BIOSIS, CABI, CCC, DIIDW, KJD, MEDLINE, RSCI, SCIELO, ZOOREC Timespan=All years*<br>*Search language=Auto*

medrxiv
“COVID-19 and antimicrobial resistance”
