# Supplementary material for: Antimicrobial resistance (AMR) in COVID-19 patients: a systematic review and meta-analysis (November 2019–June 2021)
Source: Antimicrob Resist Infect Control. 2022 Mar 7;11:45. doi: 10.1186/s13756-022-01085-z (PMC8899460; doi:10.1186/s13756-022-01085-z)
Supplement: Supplementary file 3 — Additional file 3. JBI Checklists [file 13756_2022_1085_MOESM3_ESM.pdf]

JBI Critical Appraisal Checklist for Cohort Studies (A) (n= 9 studies; 7 retrospective and 2 prospective cohorts)

	Chowdhary et al. (2020)	Ramadan et al. (2020)	Amarsy et al. (2020)	Perez et al. (2020)	Cataldo et al. (2020)	Nori et al. (2020)	Sharifipour et al. (2020)	Razazi et al. (2020)	Tiri et al. (2020)
1. Were the two groups similar and recruited from the same population?	N/A	N/A	Yes	Yes	Unclear	Unclear	No	Yes	N/A
2. Were the exposures measured similarly to assign people to both exposed and unexposed groups?	Yes	Yes	Yes	Yes	Unclear	Unclear	Yes	Yes	Yes
3. Was the exposure measured in a valid and reliable way?	Yes	Yes	Yes	Yes	Unclear	Yes	Yes	Yes	Yes
4. Were confounding factors identified?	Yes	Yes	Unclear	Yes	No	No	No	No	Yes
5. Were strategies to deal with confounding factors stated?	N/A	Yes	Unclear	No	No	No	Yes	No	Yes
6. Were the groups/participants free of the outcome at the start of the study (or at the moment of exposure)?	Yes	Yes	Yes	Yes	Unclear	Unclear	Yes	Yes	Yes

7. Were the outcomes measured in a valid and reliable way?	Yes	Yes	Yes	Yes	Yes	Yes	Yes	Yes	Yes
8. Was the follow up time reported and sufficient to be long enough for outcomes to occur?	Yes	Yes	Yes	Yes	Unclear	Yes	No	Yes	Yes
9. Was follow up complete, and if not, were the reasons to loss to follow up described and explored?	Yes	Yes	N/A	N/A	No	Yes	No	No	Yes
10. Were strategies to address incomplete follow up utilized?	N/A	N/A	N/A	N/A	No	N/A	No	N/A	N/A
11. Was appropriate statistical analysis used?	Yes	Yes	Unclear	Unclear	Unclear	Unclear	Unclear	Yes	Yes

JBI Critical Appraisal Checklist for Cohort Studies (B) (n= 9 studies; 6 retrospective and 3 prospective cohorts)

	Moretti et al. (2021)	Posterero et al. (2021)	Grasselli et al. (2021)	Baskaran et al. (2021)	Gomez-Simmonds et al. (2021)	Karruli et al. (2021)	Khurana et al. (2021)	Kokkoris et al. (2021)	Martinez-Guerra et al. (2021)
1. Were the two groups similar and recruited from the same population?	Yes	Yes	Yes	Yes	No	No	Yes	Yes	No
2. Were the exposures measured similarly to assign people to both exposed and unexposed groups?	Yes	Yes	Yes	Yes	Unclear	Unclear	Yes	Yes	Yes

3. Was the exposure measured in a valid and reliable way?	Yes	Yes	Unclear	Unclear	Yes	Yes	Yes	Yes	Yes
4. Were confounding factors identified?	Yes	Yes	Yes	Yes	No	No	No	No	No
5. Were strategies to deal with confounding factors stated?	Yes	Yes	Yes	Yes	No	No	No	No	No
6. Were the groups/participants free of the outcome at the start of the study (or at the moment of exposure)?	Yes	Yes	Yes	Yes	Yes	Yes	Yes	Yes	Yes
7. Were the outcomes measured in a valid and reliable way?	Yes	Yes	Unclear	Unclear	Yes	Yes	Yes	Yes	Yes
8. Was the follow up time reported and sufficient to be long enough for outcomes to occur?	Yes	Yes	Yes	Yes	Unclear	Unclear	Unclear	Unclear	Yes
9. Was follow up complete, and if not, were the reasons to loss to follow up described and explored?	N/A	N/A	Yes	N/A	No	No	Yes	No	No
10. Were strategies to address incomplete follow up utilized?	N/A	N/A	N/A	N/A	No	No	No	No	No
11. Was appropriate statistical analysis used?	Yes	Yes	Yes	Yes	Yes	Yes	Yes	Yes	Yes

JBI Critical Appraisal Checklist for Cohort Studies (C) (n= 3 studies; 2 retrospective and 1 prospective cohort)

	Suarez-de-la-Rica et al. (2021)	Llopis-Pastor et al. (2021)	Cultrera et al. (2021)
1. Were the two groups similar and recruited from the same population?	Yes	N/A	Yes
2. Were the exposures measured similarly to assign people to both exposed and unexposed groups?	Yes	N/A	Yes
3. Was the exposure measured in a valid and reliable way?	Yes	Yes	Yes
4. Were confounding factors identified?	No	Yes	Unclear
5. Were strategies to deal with confounding factors stated?	No	Unclear	Unclear
6. Were the groups/participants free of the outcome at the start of the study (or at the moment of exposure)?	Yes	Yes	Yes
7. Were the outcomes measured in a valid and reliable way?	Yes	Yes	Yes

8. Was the follow up time reported and sufficient to be long enough for outcomes to occur?	Unclear	Yes	Yes
9. Was follow up complete, and if not, were the reasons to loss to follow up described and explored?	No	Yes	N/A
10. Were strategies to address incomplete follow up utilized?	No	N/A	N/A
11. Was appropriate statistical analysis used?	Yes	Yes	Yes

JBI Critical Appraisal Checklist for Case Control Studies (n= 3 studies; 3 retrospective case control)

	Bogossian et al. (2020)	Baiou et al. (2021)	Bentivegna et al. (2021)
1. Were the groups comparable other than the presence of disease in cases or the absence of disease in controls?	Unclear	Yes	Yes
2. Were cases and controls matched appropriately?	Yes	Yes	Unclear
3. Was the same criteria used for identification of cases and controls?	Yes	Yes	Yes
4. Was exposure measured in a standard, valid and reliable way?	Yes	Yes	Yes
5. Was exposure measured in the same way for cases and controls?	Yes	Yes	Yes
6. Were confounding factors identified?	Yes	Yes	Yes
7. Were strategies to deal with confounding factors stated?	Yes	Yes	Yes
8. Were outcomes assessed in a standard, valid and reliable way for cases and controls?	Yes	Yes	Yes
9. Was the exposure period of interest long enough to be meaningful?	Yes	Yes	Yes
10. Was appropriate statistical analysis used?	Yes	Yes	Yes

JBI Critical Appraisal Checklist for Quasi-Experimental Studies (n= 1 study; retrospective cohort)

	Guisado-Gil et al. (2020)
1. Is it clear in the study what is the 'cause' and what is the 'effect' (i.e. there is no confusion about which variable comes first)?	Yes
2. Were the participants included in any comparisons similar?	Yes
3. Were the participants included in any comparisons receiving similar treatment/care, other than the exposure or intervention of interest?	Yes
4. Was there a control group?	Yes
5. Were there multiple measurements of the outcome both pre and post the intervention/exposure?	Yes
6. Was follow up complete and if not, were differences between groups in terms of their follow up adequately described and analyzed?	Yes
7. Were the outcomes of participants included in any comparisons measured in the same way?	Yes
8. Were outcomes measured in a reliable way?	Yes
9. Was appropriate statistical analysis used?	Yes

JBI Critical Appraisal Checklist for Case Series (n= 4 studies; retrospective observational studies)

	Mo et al. (2021)	Garcia-Meniño et al. (2020)	Montrucchio et al. (2020)	Mady et al. (2020)
1. Were there clear criteria for inclusion in the case series?	Yes	No	Yes	Yes
2. Was the condition measured in a standard, reliable way for all participants included in the case series?	Unclear	Yes	Yes	Yes
3. Were valid methods used for identification of the condition for all participants included in the case series?	Yes	Yes	Yes	Yes
4. Did the case series have consecutive inclusion of participants?	Yes	Yes	Yes	Yes
5. Did the case series have complete inclusion of participants?	Yes	Yes	Yes	No
6. Was there clear reporting of the demographics of the participants in the study?	Yes	Yes	Yes	Yes
7. Was there clear reporting of clinical information of the participants?	Yes	Yes	Yes	Yes
8. Were the outcomes or follow up results of cases clearly reported?	Yes	Yes	Yes	Yes
9. Was there clear reporting of the presenting site(s)/clinic(s) demographic information?	Yes	Yes	Yes	Yes
10. Was statistical analysis appropriate?	Yes	N/A	Yes	Yes

JBI Critical Appraisal Checklist for Case Reports (n= 3 studies)

	Posteraro et al. (2020)	Walpole et al. (2020)	Perrotta et al. (2021)
1. Were patient's demographic characteristics clearly described?	Yes	Yes	Yes
2. Was the patient's history clearly described and presented as a timeline?	Yes	N/A	No
3. Was the current clinical condition of the patient on presentation clearly described?	Yes	Yes	Yes
4. Were diagnostic tests or assessment methods and the results clearly described?	Yes	Yes	Yes
5. Was the intervention(s) or treatment procedure(s) clearly described?	Yes	Yes	Yes
6. Was the post-intervention clinical condition clearly described?	Yes	Yes	Yes
7. Were adverse events (harms) or unanticipated events identified and described?	Yes	Yes	Unclear
8. Does the case report provide takeaway lessons?	Yes	Yes	Yes

JBI Critical Appraisal Checklist for Cross Sectional Studies (n= 6 studies; 3 cross sectional studies and 3retrospective studies)

	Salehi et al. (2020)	Mahmoudi et al. (2020)	Li et al. (2020)	Contou et al. (2020)	Magnasco et al. (2021)	Pascale et al. (2021)
1. Were the criteria for inclusion in the sample clearly defined?	Yes	Yes	Yes	Yes	Yes	Yes
2. Were the study subjects and the setting described in detail?	Yes	No	Yes	Yes	Yes	Yes
3. Was the exposure measured in a valid and reliable way?	Yes	Yes	Yes	Yes	Yes	Yes
4. Were objective, standard criteria used for measurement of the condition?	Yes	Yes	Yes	Yes	Yes	Yes
5. Were confounding factors identified?	Yes	Unclear	Yes	Yes	Yes	Unclear
6. Were strategies to deal with confounding factors stated?	Yes	Unclear	Yes	Yes	Yes	Unclear
7. Were the outcomes measured in a valid and reliable way?	Yes	Yes	Yes	Yes	Yes	Yes
8. Was appropriate statistical analysis used?	Yes	Unclear	Yes	Unclear	Unclear	Yes
