# Supplementary material for: Antimicrobial resistance (AMR) in COVID-19 patients: a systematic review and meta-analysis (November 2019–June 2021)
Source: Antimicrob Resist Infect Control. 2022 Mar 7;11:45. doi: 10.1186/s13756-022-01085-z (PMC8899460; doi:10.1186/s13756-022-01085-z)
Supplement: Supplementary file 4 — Additional file 4. QUADAS 2 Checklist [file 13756_2022_1085_MOESM4_ESM.pdf]

QUADAS 2 CHECKLISTS – Domain 3: reference standard (A)

	Chowdhary et al. (2020)	Bogossian et al. (2020)	Ramadan et al. (2020)	Amarsy et al. (2020)	Perez et al. (2020)	Salehi et al. (2020)	Cataldo et al. (2020)
**A. Risk of Bias**							
Describe the reference standard and how it was interpreted	Isolates were identified by matrix-assisted laser desorption/ionization time-of-flight mass spectrometry (MALDI Biotyper, https://www.bruker.com). In addition, species identification was conducted by amplification and sequencing of the internal transcribed spacer region of ribosomal DNA and of the D1/D2 domain of the large subunit ribosomal DNA. Antifungal susceptibility testing was performed by using the Clinical and Laboratory	Routine surveillance cultures (rectal swab, tracheal aspirate, urinary cultures) assessed for microbiology. Rectal swabs streaked using chromID CARBA SMART agar for the detection of CPE, MacConkey agar with ceftazidime for the detection of 3rd generation cephalosporin-resistant *P. aeruginosa*, *Klebsiella* and *Enterobacter spp*, chromID CRE agar for the detection of VRE and MALDI-TOF. AMR was defined by breakpoints according to EUCAST,	Bacterial DNA was extracted from the clinical isolates obtained from COVID-19 co-infected patients using QIAamp® DNA Mini Kit (QIAGEN GmbH, Hilden, Germany) in accordance with manufacturer's instructions and stored at −20 °C. Monoplex PCR assay was performed to identify and amplify the following antimicrobial resistance genes (mecA, NDM-1, KPC, TEM, CTX-M, and SHV) obtained from multidrug-resistant isolated strains.	Blood and respiratory specimen cultures positive for Serratia marcescens, resistant to amoxicillin, amoxiclav, 1st and 2nd generation cephalosporins (inducible AmpC -lactamase) and with lowlevel resistance to amikacin (chromosome-borne aac(6')-Ic). Isolates were clonal based on whole genome sequencing using IlluminaTM procedures.	Incident cases were identified from clinical specimens and through colonization screening. The multidrug-resistant CRAB definition (*A. baumannii* with documented resistance to three or more classes of antibiotics) was applied to hospital clinical laboratory antimicrobial susceptibility data for incident cases. Isolates were further evaluated for carbapenemase genes through real-time polymerase chain reaction testing. Testing was performed at the Clinical	Oropharyngeal candidiasis was confirmed by the presence of budding yeasts and pseudohyphae in KOH 10% preparation and culture. Antifungal susceptibility patterns of isolates to three classes of antifungal drugs, i.e. azoles (fluconazole, voriconazole, and itraconazole), polyenes (amphotericin B), and echinocandins (caspofungin, anidulafungin, and micafungin) were assessed according to the fourth edition of the Clinical and Laboratory Standards	Incidence of BSIs/10000ICU days was calculated including only the first episode of BSI. Subsequent BSIs acquired after the first episode of BSIs were also collected. Aetiology of BSI and susceptibility patterns of the isolates were recorded. Rate of BSIs inpatients admitted to our ICU in the first 6 months of the previous year were obtained. Results of surveillance samples of the rectum to detect colonisation by MDR organisms were also collected.

	Standards Institute broth-microdilution method M27-A3/S4	VITEK2 and disk diffusion. Carbapanemases OXA-48, KPC, NDM, VIM and IMP detected through PCR or Coris Resist-5 O.O.K.N.V antigenic detection VanA and VanB genes detected through PCR. ESBL and AmpC identified through synergy of disk diffusion through EUCAST. MRSA: ChromID MRSA selective plates. MDR *Pseudomonas* and *Acinetobacter* defined and recommended considering antimicrobial resistant phenotype.			Laboratory Improvement Amendments (CLIA)– certified Northeast Regional Antimicrobial Resistance Laboratory located at the Wadsworth Center at the David Axelrod Institute in Albany, New York	Institute M27 standard method. The minimum inhibitory concentrations (MICs) were interpreted according to the breakpoints or epidemiological cut-off values (ECV) provided in CLSI M60 and M59 supplements	
❖ Is the reference standard likely to correctly classify the target condition?	Yes	Yes	Yes	Unclear	Yes	Yes	Unclear
❖ Were the reference standard results interpreted without knowledge of the results of the index test?	Yes	Yes	Unclear	Unclear	Yes	Yes	Unclear

Could the reference standard, its conduct, or its interpretation have introduced bias?	Low	Low	Low	Unclear	Low	Low	Unclear
**B. Concerns regarding applicability**							
Is there concern that the target condition as defined by the reference standard does not match the review question?	Low	Low	Low	Unclear	Low	Low	Unclear

QUADAS 2 CHECKLISTS – Domain 3: reference standard (B)

	Posteraro et al. (2020)	Nori et al. (2020)	Mahmoudi et al. (2020)	Li et al. (2020)	Contou et al. (2020)	Mo et al. (2021)	Garcia-Meniño et al. (2020)
**A. Risk of Bias**							
Describe the reference standard and how it was interpreted	Blood cultures yielded a yeast organism, later identified as *C. glabrata* using a previously described matrix-assisted laser desorption/ionization time-of-flight (MALDI-TOF) mass spectrometry-based method. Only for echinocandin	All adult and pediatric patients with a positive SARS-CoV-2 PCR result and positive blood or respiratory culture (by matrix-assisted laser desorption/ionization) were analyzed. Cases were included if the positive PCR result and microbiology	BC and ETA cultures were obtained from COVID-19 patients. Swabs and blood were cultured on blood agar and MacConkey agar plates and incubated at 37°C for 18–24 hours. Identification of the isolated bacteria was performed using	At least one positive etiology of bacteria was acquired from qualified microbiological specimens (qualified sputum, endotracheal aspirate, bronchoalveolar lavage fluid, blood samples, or qualified urine). Susceptibility testing was	Blood cultures, cultures of the respiratory tract secretions, multiplex respiratory PCRs Panel RP2 plus (Film Array Biomerieux®), Panel Pneumonia Plus (Film Array Biomerieux®) performed on a nasopharyngeal swab or on respiratory tract	Neither method of organism identification nor antimicrobial susceptibility testing method were described.	Bacterial identification was performed by MALDI TOF/MS (Bruker Daltonics, Bremen, Germany) and antimicrobial susceptibility testing carried out by the Microscan System (Beckman Coulter, Brea, CA, USA), and the results

	antifungal agents, MIC values obtained with the SensititreYeastOn e® method were confirmed by the CLSI M27-A3 reference method.	result occurred in the same or preceding admission (within 30 days)	standard microbiological methods. For all isolated strains, antibacterial susceptibility was tested using the standard Kirby-Bauer disk-diffusion method on Mueller Hinton agar (Merk Co., Germany) in accordance with the CLSI guidelines (CLSI; 2019, M100-S29).	carried out on the Phoenix-100 automatic microbiological system, KirbyBauer method was also used. Results were interpreted according to the criteria of the Clinical and Laboratory Standards Institute.	secretions, urinary antigen tests (BinaxNOW®-Abbott) for *Legionella pneumophila* and *Streptococcus pneumoniae*.		interpreted according to EUCAST (www.eucast.org). Bacterial DNA was extracted by boiling lysis method and extended-spectrum-lactamase (ESBL) and carbapenemase encoding genes were screened by the AMR Flow Chip system (MásterDiagnós-tica, Granada, Spain), conventional PCR and further sequencing as previously described.
❖ Is the reference standard likely to correctly classify the target condition?	Yes	Yes	Yes	Yes	Unclear	Unclear	Yes
❖ Were the reference standard results interpreted without knowledge of the results of the index test?	Yes	Yes	Yes	Yes	Unclear	Unclear	Yes
Could the reference standard, its conduct, or its interpretation have introduced bias?	Low	Low	Low	Low	Unclear	Unclear	Low

**B. Concerns regarding applicability**							
Is there concern that the target condition as defined by the reference standard does not match the review question?	Low	Low	Low	Low	Unclear	Unclear	Low

QUADAS 2 CHECKLISTS – Domain 3: reference standard (C)

	Sharifipour et al. (2020)	Walpole et al. (2020)	Razazi et al. (2020)	Guisado-Gil et al. (2020)	Montrucchio et al. (2020)	Mady et al. (2020)	Tiri et al. (2020)
**A. Risk of Bias**							
Describe the reference standard and how it was interpreted	AST was separately performed on isolated bacteria at each stage of sampling and was evaluated by the standard disc diffusion method in accord with the recommendations of the Clinical & Laboratory Standards Institute or CLSI	Sputum culture grew *Y. enterocolitica*, resistant to amoxicillin and co-amoxiclav but otherwise fully sensitive.	Bacterial coinfection at ICU admission was evidenced by the detection of bacteria in the sputum or in blood samples, in the absence of other sources of infection, or by a positive pneumococcal or *L. pneumophila* serotype 1 urinary antigen test	BSI diagnosed by blood cultures obtained > 48 hours after admission. AST based on EUCAST criteria, and MDR categorization fulfilled German Society for Hygene and Microbiology.	MALDI-ToF MS, Microscan WalkAway plus System, MASTDISCS Combi Carba plus disk for ID. AST using Xpert Carba-R assay and interpreted by EUCAST	VAP: respiratory culture positive or PCR; BSI: blood culture positive; UTI: urinary cultures. AST information not provided.	Screening for colonization of CRE through rectal swabbing or clinical culture (BAL or UCx) and identification through Vitek-MS. AST tested using Vitek2 and immunochromatography for OXA-48 OXA-163, KPC, NDM, VIM.

❖ Is the reference standard likely to correctly classify the target condition?	Yes	Unclear	Unclear	Yes	Yes	Unclear	Yes
❖ Were the reference standard results interpreted without knowledge of the results of the index test?	Yes	Unclear	Unclear	Yes	Yes	Unclear	Yes
Could the reference standard, its conduct, or its interpretation have introduced bias?	Low	Unclear	Unclear	Low	Low	Unclear	Low
**B. Concerns regarding applicability**							
Is there concern that the target condition as defined by the reference standard does not match the review question?	Low	Unclear	Unclear	Low	Low	Unclear	Low

QUADAS 2 CHECKLISTS – Domain 3: reference standard (D)

	Baiou et al. (2021)	Moretti et al. (2021)	Posterero et al. (2021)	Grasselli et al. (2021)	Baskaran et al. (2021)	Magnasco et al. (2021)	Gomez-Simmonds et al. (2021)
**A. Risk of Bias**							

Describe the reference standard and how it was interpreted	Matrix-assisted laser desorption/ionization-time of flight (MALDI-TOF) mass spectrometry was used for bacterial identification. BD Phoenix was used for antimicrobial susceptibility testing and for the detection of extended-spectrum beta-lactamases (ESBLs). Clinical Laboratory Standards Institute breakpoints were used.	Five Klebsiella pneumoniae and one by Klebsiella oxytoca were classified as ESBL following the criteria European committee on antimicrobial susceptibility testing (EUCAST).	For BSI isolates, identification was performed using the MALDI Biotyper® system, and antimicrobial susceptibility testing (AST) was performed using VITEK® 2 or, only for Candida isolates, Sensititre™ YeastOne™ systems. EUCAST clinical breakpoints were used to interpret minimum inhibitory concentration (MIC) values to all routinely tested antimicrobial drugs, except for those of fluconazole or echinocandin antifungal drugs that were interpreted according to clinical breakpoints reported in the CLSI M27-S4 document.	Infections were identified and recorded considering all microbiologic isolates obtained during the ICU course, independently reviewed and classified in light of the available clinical, laboratory, and radiographic data by dedicated intensivists (one for each center) and infectious disease specialists (one for each center), following international guidelines.	Diagnostic microbiology tests were performed as per standard testing protocols within NHS laboratories at individual participating sites. Microbiology results included in the analysis were: standard culture (blood, sputum, trachealaspirate, bronchoalveolar lavage (BAL), urine) and validated culture-independent tests such as respiratory viral PCR and urinary antigens.	Microorganism cultured from blood, respiratory or urinary samples were identified using MALDI-TOF mass spectrometry. Antimicrobial susceptibility testing was performed using the Vitek2 system, while antifungal testing was carried out by means of the Clinical and Laboratory Standards Institute microdilution method and the Sensititre YeastOne panel. *C. auris* clonal relatedness was evaluated by means of whole genome sequencing.	Clinical isolates underwent routine species identification and susceptibility testing by MicroScan (Beckman Coulter) in our clinical micro-biology laboratory. For isolates belonging to Klebsiella pneumoniaeST258, we constructed phylogenetic trees from isolates with available nanopore sequencing data.

❖ Is the reference standard likely to correctly classify the target condition?	Yes	Yes	Yes	Yes	Unclear	Yes	Unclear
❖ Were the reference standard results interpreted without knowledge of the results of the index test?	Yes	Yes	Yes	Yes	Unclear	Yes	Unclear
Could the reference standard, its conduct, or its interpretation have introduced bias?	Low	Low	Low	Low	Unclear	Low	Low
**B. Concerns regarding applicability**							
Is there concern that the target condition as defined by the reference standard does not match the review question?	Low	Low	Low	Low	Unclear	Low	Low

QUADAS 2 CHECKLISTS – Domain 3: reference standard (E)

	Karruli et al. (2021)	Kokkoris et al. (2021)	Perrotta et al. (2021)	Martinez-Guerra et al. (2021)	Suarez-de-la-Rica et al. (2021)	Khurana et al. (2021)	Llopis-Pastor et al. (2021)

A. Risk of Bias							
Describe the reference standard and how it was interpreted	Hematochemical parameters were recorded on ICU admission and on the day of microbiological sampling showing positivity for MDR pathogen or, for those without MDR infections, after 3–8 days dwelling in ICU, and included white blood cells and differential, platelet count, C-reactive protein, creatinine, bilirubin, international normalized ratio, and activated partial thromboplastin time. Infections were diagnosed based on the current U.S. Centers for Disease Control and Prevention National Health. care Safety Net-work criteria.	ICU-acquired BSI was defined as a pathogen isolation from1blood specimen obtained at more than 48 h after ICU admission. In patients with greater than or equal to 2 BSIs, only the first one was included, unless the subsequent episode was fungal. Overall, 50 episodes of ICU-acquired BSIs were registered. (No other information about microbiological techniques was provided in this article)	A CPE surveillance rectal swab performed on admission in the ICU tested positive for Klebsiella Pneumoniae NDM.	Bacterial isolates identification was done through Bruker Microflex LT, using Bruker Biotyper software. When the MALDI-Biotyper did not identify the isolate, we used Vitek2 system cards for Gram-positive and Gram-negative organisms, following the manufacturer's instructions.	Both endotracheal aspirates and blood cultures were requested by the attending physician in case of clinical suspicion with radiological and/or laboratory findings. Patients with no blood or respiratory cultures were considered not to have secondary infections	The BioFire® Film Array® Respiratory (RP) Panel was used for detection of co-infection with 15 bacteria&20 other respiratory pathogens including17 viruses; Adenovirus, Coronaviruses (HCoV-HKU, HCoV-NL63, HCoV-229E, HCoVOC43), Human Metapneumovirus (HMPV), Human Rhinovirus/Entero virus (HRV), Influenza viruses (FluA, FluA/H, FluA/H3, FluA/H1-2009, and FluB), Parainfluenza types (PIV 1, 2, 3,&4), and Respiratory Syncytial Virus (RSV), and 3 atypical bacteria including Bordetella pertussis, Chlamydia pneumoniae, and Mycoplasma pneumoniae, only in the respiratory samples of	Not described.

						COVID-19 patients	
❖ Is the reference standard likely to correctly classify the target condition?	Yes	Unclear	Unclear	Yes	Unclear	Yes	Unclear
❖ Were the reference standard results interpreted without knowledge of the results of the index test?	Unclear	Unclear	Unclear	Unclear	Unclear	Unclear	Unclear
Could the reference standard, its conduct, or its interpretation have introduced bias?	Low	Unclear	Unclear	Low	Unclear	Low	Unclear
**B. Concerns regarding applicability**							
Is there concern that the target condition as defined by the reference standard does not match the review question?	Low	Unclear	Unclear	Low	Unclear	Low	Unclear

QUADAS 2 CHECKLISTS – Domain 3: reference standard (F)

	Pascale et al. (2021)	Bentivegna et al. (2021)	Cultrera et al. (2021)
**A. Risk of Bias**			
Describe the reference standard and how it was interpreted	CPE strains collected in accordance to active surveillance and screening guidelines	Not described	MRSA and VRE strains were detected by cefoxitin screening and an oxacillin MIC test with Card AST-P659 and a teicoplanin/vancomicin MIC test with Card AST-P658, respectively. MIC values were interpreted according to current EUCAST clinical breakpoints. CPE strains were confirmed by microdilution (Sensititre™, Thermo Fisher Scientific) EURGNCOL and DKMGN plates. Phenotypical CPE resistance was confirmed by synergic test diffusion Diatabs™ (Rosco

			Diagnostica) on Mueller–Hinton agar (Vacutest-Kima). A genotyping test for CPE resistance was performed by RT-PCR (GeneXpert®) with an Xpert® Carba-R test. MIC values were interpreted according to current EUCAST clinical breakpoints.
❖ Is the reference standard likely to correctly classify the target condition?	Unclear	Unclear	Yes
❖ Were the reference standard results interpreted without knowledge of the results of the index test?	Unclear	Unclear	Yes
Could the reference standard, its conduct, or its interpretation have introduced bias?	Unclear	Unclear	Low
**B. Concerns regarding applicability**			

Is there concern that the target condition as defined by the reference standard does not match the review question?	Unclear	Unclear	Low
