# Supplementary material for: Antimicrobial resistance (AMR) in COVID-19 patients: a systematic review and meta-analysis (November 2019–June 2021)
Source: Antimicrob Resist Infect Control. 2022 Mar 7;11:45. doi: 10.1186/s13756-022-01085-z (PMC8899460; doi:10.1186/s13756-022-01085-z)
Supplement: Supplementary file 8 — Additional file 8. Patient level data [file 13756_2022_1085_MOESM8_ESM.docx]

**Patient level data**

| Study | Author | Patient | Age | Sex | Clinical Disease | Comorbidities | Co-Infecting Organism | Resistant | Resistance Profile | Mechanically Ventilated | Days of MV | Antibiotics prior to diagnosis | COVID-19 Therapy | Outcome |
| --- | --- | --- | --- | --- | --- | --- | --- | --- | --- | --- | --- | --- | --- | --- |
| 1 | Walpole | 1 | 33 | M | COVID-19: presenting as severe, persistent abdominal pain | No | Yersinia enterocolitica | Yes | amoxicillin, co-amoxicillin | No | NA | Pip-Tazo | None | Alive |
| 2 | Montrucchio | 1 | 50 | M | ARDS | No | Klebsiella pneumoniae | Yes | OXA-48 producing KP | Yes | 24 | Ceftazidime/Avibactam + Foscomycin + TMP/SMX | Darunavir/Ritonavir + HCQ + steroids | Dead |
| 2 | Montrucchio | 2 | 41 | M | ARDS | Yes | Klebsiella pneumoniae | Yes | OXA-48 producing KP | Yes | 46 | Ceftazidime/Avibactam + TMP/SMX | Lopinavir/ritonavir + HCQ + steroids + redemsivir | Alive |
| 2 | Montrucchio | 3 | 54 | F | ARDS | Yes | Klebsiella pneumoniae | Yes | OXA-48 producing KP | Yes | 34 | Ceftazidime/Avibactam + Colistin | DRV/Ciobicistat + HCQ + steroids + tocilizumab | Alive |
| 2 | Montrucchio | 4 | 62 | F | ARDS | Yes | Klebsiella pneumoniae | Yes | OXA-48 producing KP | Yes | 37 | Ceftazidime/Avibactam | HCQ + steroids + tocilizumab + redemsivir | Alive |
| 2 | Montrucchio | 5 | 71 | M | ARDS | Yes | Klebsiella pneumoniae | Yes | OXA-48 producing KP | Yes | 20 | None | HCQ | Alive |
| 2 | Montrucchio | 6 | 52 | M | ARDS | Yes | Klebsiella pneumoniae | Yes | OXA-48 producing KP | Yes | 42 | Meropenem + Ertapenem + Tigecycline | HCQ + steroids + tocilizumab | Alive |
| 2 | Montrucchio | 7 | 67 | F | ARDS | Yes | Klebsiella pneumoniae | Yes | OXA-48 producing KP | Yes | 21 | Ceftazidine/Avibatam + Fosfomycin | HCQ + steroids + tocilizumab + redemsivir | Dead |
| 3 | Mo | 1 | 45 | M | COVID-19 + pneumonia | No | K. pneumoniae | Yes | ESBL | NA | NA | None | Tocilizumab | Dead |
| 3 | Mo | 2 | 45 | F | COVID-19 + pneumonia + bacteremia | Yes | P. aeruginosa, C. auris, VRE | Yes | MDR P. aeruginosa, VRE | NA | NA | None | Tocilizumab | Alive |
| 3 | Mo | 3 | 44 | M | COVID-19 + pneumonia | No | MRSA | Yes | MRSA | NA | NA | Azithromycin | Tocilizumab | Alive |
| 3 | Mo | 4 | 73 | F | COVID-19 + pneumonia | Yes | P. aeruginosa | Yes | MDR | NA | NA | Azithromycin | Tocilizumab | Dead |
| 3 | Mo | 5 | 63 | F | COVID-19 + pneumonia + UTI | Yes | A. baumanii, K. pneumoniae | Yes | MDR + ESBL | NA | NA | Azithromycin | Tocilizumab | Dead |
| 3 | Mo | 6 | 45 | F | COVID-19 + wound infection | Yes | S. maltiphila | Yes | MDR | NA | NA | Azithromycin | Tocilizumab | Alive |
| 3 | Mo | 7 | 51 | F | COVID-19 + pneumonia | No | K. oxytoca | Yes | ESBL + | NA | NA | Azithromycin | Tocilizumab | Alive |
| 3 | Mo | 8 | 71 | F | COVID-19 + pneumomia + UTI | Yes | MRSA, C. albicans | Yes | MRSA | NA | NA | Azithromycin | Tocilizumab | Alive |
| 3 | Mo | 9 | 47 | M | COVID-19 + pneumomia | Yes | A. baumanii, K. pneumoniae | No | None | NA | NA | Azithromycin | Tocilizumab | Dead |
| 3 | Mo | 10 | 63 | M | COVID-19 + pneumomia | Yes | K. pneumoniae | No | None | NA | NA | Azithromycin | Tocilizumab | Alive |
| 3 | Mo | 11 | 68 | F | COVID-19 + wound infection | Yes | MRSA | Yes | MRSA | NA | NA | Azithromycin | Tocilizumab | Dead |
| 4 | Posteraro | 1 | 79 | M | COVID-19 + BSI | Yes | C. glabrata | Yes | pan-echinocandin resistance | No | NA | Pip-Tazo | Darunavir/Ritonavir + HCQ | Dead |
| 5 | Chowdhary | 1 | 25 | F | COVID-19 + candidemia | Yes | C. auris | Yes | Fluconazole | No | NA | AMB, Azithromycin | HCQ | Alive |
| 5 | Chowdhary | 2 | 52 | M | COVID-19 + candidemia | Yes | C. auris | Yes | Fluconazole | Yes | NA | MFG. AMB, Azithromycin | HCQ, Tocilizumab, convalescent plasma | Dead |
| 5 | Chowdhary | 3 | 82 | F | COVID-19 + candidemia | Yes | C. auris | Yes | Fluconazole | Yes | NA | MFG, Azithromycin | HCQ + Favipiravir | Dead |
| 5 | Chowdhary | 4 | 86 | F | COVID-19 + candidemia | Yes | C. auris | Yes | Fluconazole | No | NA | MFG, Azithromycin | HQQ + Redemsivir | Dead |
| 5 | Chowdhary | 5 | 66 | M | COVID-19 + candidemia | Yes | C. auris | Yes | Fluconazole | No | NA | MFG. AMB, Azithromycin | Redemsivir | Alive |
| 5 | Chowdhary | 6 | 71 | M | COVID-19 + candidemia | Yes | C. auris | Yes | Fluconazole | Yes | NA | MFG, Azithromycin | Falvipiravir | Dead |
| 5 | Chowdhary | 7 | 67 | M | COVID-19 + candidemia | Yes | C. auris | Yes | Fluconazole | No | NA | MFG. AMB, Azithromycin | Redemsivir, convalescent plasma | Alive |
| 5 | Chowdhary | 8 | 72 | M | COVID-19 + candidemia | Yes | C. auris | Yes | Fluconazole | Yes | NA | MFG, Azithromycin | Falcipiravir, Tocilizumab, convalescent plasma | Dead |
| 5 | Chowdhary | 9 | 81 | M | COVID-19 + candidemia | Yes | C. auris | Yes | Fluconazole | No | NA | MFG, Azithromycin | Falcipiravir | Dead |
| 5 | Chowdhary | 10 | 69 | M | COVID-19 + candidemia | Yes | C. auris | Yes | Fluconazole | Yes | NA | MFG, Azithromycin | Favipiravir, Tocilizumab, convalescent plasma |  |
| 6 | Garcia-Menino | 1 | 66 | M | COVID-19 + BSI | No | K. pneumoniae | Yes | CRE (CTX-M-15 anmd OXA-48 producing KP belonging to ST326) | Yes | NA | Yes | Yes | Alive |
| 6 | Garcia-Menino | 2 | 67 | M | Colonization | Yes | K. pneumoniae | Yes | CRE | Yes | NA | Yes | Yes | Alive |
| 6 | Garcia-Menino | 3 | 76 | M | Infection | Yes | K. pneumoniae | Yes | CRE | Yes | NA | Yes | Yes | Dead |
| 6 | Garcia-Menino | 4 | 69 | M | Infection | No | K. pneumoniae | Yes | CRE | Yes | NA | Yes | Yes | Alive |
| 6 | Garcia-Menino | 5 | 71 | F | Colonization | Yes | K. pneumoniae | Yes | CRE | Yes | NA | Yes | Yes | Alive |
| 6 | Garcia-Menino | 6 | 54 | M | Colonization | Yes | K. pneumoniae | Yes | CRE | Yes | NA | Yes | Yes | Alive |
| 6 | Garcia-Menino | 7 | 66 | M | Colonization | Yes | K. pneumoniae | Yes | CRE | Yes | NA | Yes | Yes | Alive |
| 7 | Sharifipour | 1 | NA | NA | VAP | Yes | A. baumanii | Yes | except colistin | Yes | NA | Ceftriaxone and Azithromycin | NA | Died |
| 7 | Sharifipour | 2 | NA | NA | VAP | Yes | A. baumanii | Yes | except colistin | Yes | NA | Ceftriaxone and Azithromycin | NA | Died |
| 7 | Sharifipour | 3 | NA | NA | VAP | Yes | A. baumanii | Yes | except colistin | Yes | NA | Ceftriaxone and Azithromycin | NA | Died |
| 7 | Sharifipour | 4 | NA | NA | VAP | Yes | A. baumanii | Yes | except colistin | Yes | NA | Ceftriaxone and Azithromycin | NA | Died |
| 7 | Sharifipour | 5 | NA | NA | VAP | No | A. baumanii | Yes | except colistin | Yes | NA | Ceftriaxone and Azithromycin | NA | Died |
| 7 | Sharifipour | 6 | NA | NA | VAP | Yes | A. baumanii | Yes | except colistin | Yes | NA | Ceftriaxone and Azithromycin | NA | Died |
| 7 | Sharifipour | 7 | NA | NA | VAP | Yes | A. baumanii | Yes | except colistin | Yes | NA | Ceftriaxone and Azithromycin | NA | Died |
| 7 | Sharifipour | 8 | NA | NA | VAP | Yes | A. baumanii | Yes | except colistin | Yes | NA | Ceftriaxone and Azithromycin | NA | Died |
| 7 | Sharifipour | 9 | NA | NA | VAP | Yes | A. baumanii | Yes | except colistin | Yes | NA | Ceftriaxone and Azithromycin | NA | Died |
| 7 | Sharifipour | 10 | NA | NA | VAP | No | MSSA | No | No | Yes | NA | Ceftriaxone and Azithromycin | NA | Alive |
| 7 | Sharifipour | 11 | NA | NA | VAP | No | A. baumanii | Yes | except colistin | Yes | NA | Ceftriaxone and Azithromycin | NA | Died |
| 7 | Sharifipour | 12 | NA | NA | VAP | Yes | A. baumanii | Yes | except colistin | Yes | NA | Ceftriaxone and Azithromycin | NA | Died |
| 7 | Sharifipour | 13 | NA | NA | VAP | Yes | A. baumanii | Yes | except colistin | Yes | NA | Ceftriaxone and Azithromycin | NA | Died |
| 7 | Sharifipour | 14 | NA | NA | VAP | Yes | A. baumanii | Yes | except colistin | Yes | NA | Ceftriaxone and Azithromycin | NA | Died |
| 7 | Sharifipour | 15 | NA | NA | VAP | Yes | A. baumanii | Yes | except colistin | Yes | NA | Ceftriaxone and Azithromycin | NA | Died |
| 7 | Sharifipour | 16 | NA | NA | VAP | Yes | A. baumanii | Yes | except colistin | Yes | NA | Ceftriaxone and Azithromycin | NA | Died |
| 7 | Sharifipour | 17 | NA | NA | VAP | Yes | A. baumanii | Yes | except colistin | Yes | NA | Ceftriaxone and Azithromycin | NA | Died |
| 7 | Sharifipour | 18 | NA | NA | VAP | Yes | MRSA | Yes | MRSA | Yes | NA | Ceftriaxone and Azithromycin | NA | Died |
| 7 | Sharifipour | 19 | NA | NA | VAP | Yes | A. baumanii | Yes | except colistin | Yes | NA | Ceftriaxone and Azithromycin | NA | Died |
| 8 | Magnasco | 1 | 70 | M | No | Yes | C. auris | Yes | Amphotericin B | NA | NA | 5th generation cephalosporin | NA | Died |
| 8 | Magnasco | 2 | 52 | M | Lung abscess | Yes | P. aeruginosa | Yes | Carbapenem | NA | NA | 5th generation cephalosporin | NA | Alive |
| 8 | Magnasco | 3 | 51 | M | VAP | Yes | P. aeruginosa | Yes | Carbapenem | NA | NA | 5th generation cephalosporin | NA | Alive |
| 8 | Magnasco | 4 | 57 | F | VAP | Yes | P. aeruginosa | Yes | Carbapenem | NA | NA | 5th generation cephalosporin | NA | Died |
| 8 | Magnasco | 5 | 67 | M | BSI | Yes | P. aeruginosa | Yes | Carbapenem | NA | NA | 5th generation cephalosporin | NA | Alive |
| 8 | Magnasco | 6 | 62 | M | BSI, pleural empyema, lung abscess | No | P. aeruginosa, C. auris | Yes | Carbapenem, Amphotericin B | NA | NA | 5th generation cephalosporin | NA | Alive |
| 8 | Magnasco | 7 | 69 | M | BSI, lung abscess | Yes | P. aeruginosa, C. auris | Yes | Carbapenem, Amphotericin B | NA | NA | 5th generation cephalosporin | NA | Died |
| 8 | Magnasco | 8 | 50 | M | VAP | No | P. aeruginosa, C. auris | Yes | Carbapenem, Amphotericin B | NA | NA | 5th generation cephalosporin | NA | Alive |
| 8 | Magnasco | 9 | 66 | M | VAP | Yes | K. pneumoniae | Yes | Carbapenem | NA | NA | 5th generation cephalosporin | NA | Alive |
| 8 | Magnasco | 10 | 62 | M | VAP, BSI | Yes | P. aeruginosa, C. auris | Yes | Carbapenem, Amphotericin B | NA | NA | 5th generation cephalosporin | NA | Alive |
| 8 | Magnasco | 11 | 64 | M | BSI, lung abscess | Yes | P. aeruginosa, C. auris | Yes | Carbapenem, Amphotericin B | NA | NA | 5th generation cephalosporin | NA | Died |
| 8 | Magnasco | 12 | 65 | M | BSI | Yes | P. aeruginosa | Yes | Carbapenem | NA | NA | 5th generation cephalosporin | NA | Alive |
| 8 | Magnasco | 13 | 63 | M | VAP | Yes | P. aeruginosa, K. pneumoniae | Yes | Carbapenems | NA | NA | 5th generation cephalosporin | NA | Died |
| 8 | Magnasco | 14 | 67 | F | No | Yes | P. aeruginosa | Yes | Carbapenem | NA | NA | 5th generation cephalosporin | NA | Died |
| 9 | Pascale | 1 | NA | NA | No | NA | A. baumanii | Yes | Carbapenem | Yes | NA | NA | Steroids, Azithromycin, Redemsivir | Died |
| 9 | Pascale | 2 | NA | NA | No | NA | A. baumanii | Yes | Carbapenem | Yes | NA | NA | Hydroxychloroquine/chloroquine, Redemsivir, Heparin, Steroids, Tocilizumab, Azithromycin | Alive |
| 9 | Pascale | 3 | NA | NA | Pneumonia | NA | A. baumanii | Yes | Carbapenem | Yes | NA | NA | Hydroxychloroquine/chloroquine, Redemsivir, Heparin, Steroids, Tocilizumab, Azithromycin | Alive |
| 9 | Pascale | 4 | NA | NA | No | NA | A. baumanii | Yes | Carbapenem | Yes | NA | NA | Steroids, Azithromycin, Redemsivir | Died |
| 9 | Pascale | 5 | NA | NA | No | NA | A. baumanii | Yes | Carbapenem | Yes | NA | NA | Hydroxychloroquine/chloroquine, Redemsivir, Heparin, Steroids, Tocilizumab | Alive |
| 9 | Pascale | 6 | NA | NA | Pneumonia | NA | A. baumanii | Yes | Carbapenem | Yes | NA | NA | Hydroxychclroquine/chloroquine, redemsivir, azithromycin, heparin | Alive |
| 9 | Pascale | 7 | NA | NA | Pneumonia | NA | A. baumanii | Yes | Carbapenem | Yes | NA | NA | Hydroxychloroquine/chloroquine, heparin, tocilizumab, steroids, azithromycin | Died |
| 9 | Pascale | 8 | NA | NA | No | NA | A. baumanii | Yes | Carbapenem | Yes | NA | NA | Hydroxychloroquine/chloroquine, redemsivir, heparin, tocilizumab, steroids | Alive |
| 9 | Pascale | 9 | NA | NA | Pneumonia, BSI | NA | A. baumanii | Yes | Carbapenem | Yes | NA | NA | Hydroxychloroquine/chloroquine, redemsivir, heparin, tocilizumab, steroids | Died |
| 9 | Pascale | 10 | NA | NA | Pneumonia | NA | A. baumanii | Yes | Carbapenem | Yes | NA | NA | Hydroxychloroquine/chloroquine, redemsivir, heparin, tocilizumab, azithromycin | Died |
| 9 | Pascale | 11 | NA | NA | No | NA | A. baumanii | Yes | Carbapenem | Yes | NA | NA | Hydroxychloroquine/chloroquine, tocilizumab, steroids, azithromycin | Died |
| 9 | Pascale | 12 | NA | NA | No | NA | A. baumanii | Yes | Carbapenem | Yes | NA | NA | Hydroxychloroquine/chloroquine, Redemsivir, Tocilizumab, Azithromycin | Died |
| 9 | Pascale | 13 | NA | NA | No | NA | A. baumanii | Yes | Carbapenem | Yes | NA | NA | Hydroxychloroquine/chloroquine, Heparin, Tocilizumab | Alive |
| 9 | Pascale | 14 | NA | NA | No | NA | A. baumanii | Yes | Carbapenem | Yes | NA | NA | Hydroxychloroquine/chloroquine, Redemsivir, Tocilizumab | Alive |
| 9 | Pascale | 15 | NA | NA | Pneumonia | NA | A. baumanii | Yes | Carbapenem | Yes | NA | NA | Heparin, Steroids, Azithroycin | Alive |
| 9 | Pascale | 16 | NA | NA | Pneumonia | NA | A. baumanii | Yes | Carbapenem | Yes | NA | NA | Hydroxychloroquine/chloroquine, tocilizumab, steroids, azithromycin | Died |
| 9 | Pascale | 17 | NA | NA | No | NA | A. baumanii | Yes | Carbapenem | Yes | NA | NA | Hydroxychloroquine/chloroquine, Heparin, Tocilizumab | Alive |
| 9 | Pascale | 18 | NA | NA | No | NA | A. baumanii | Yes | Carbapenem | Yes | NA | NA | Heprin, Tocilizumab | Alive |
| 9 | Pascale | 19 | NA | NA | No | NA | A. baumanii | Yes | Carbapenem | Yes | NA | NA | Hydroxychloroquine/chloroquine, Heparin | Alive |
| 9 | Pascale | 20 | NA | NA | No | NA | A. baumanii | Yes | Carbapenem | Yes | NA | NA | Heparin, Tocilizumab, Steroids | Died |
| 9 | Pascale | 21 | NA | NA | No | NA | A. baumanii | Yes | Carbapenem | Yes | NA | NA | Hydroxychloroquine/chloroquine, Heparin, Tocilizumab | Alive |
| 10 | Gomez-Simmonds | 1 | 67 | M | severe COVID-19 | NA | K. pneumoniae | Yes | KPC-2 | NA | NA | NA | NA | Died |
| 10 | Gomez-Simmonds | 2 | 50 | M | severe COVID-19 | NA | K. pneumoniae | Yes | KPC-2 | NA | NA | NA | NA | Died |
| 10 | Gomez-Simmonds | 3 | 70 | M | severe COVID-19 | NA | K. pneumoniae | Yes | KPC-2 | NA | NA | NA | NA | Died |
| 10 | Gomez-Simmonds | 4 | 72 | M | severe COVID-19 | NA | K. pneumoniae | Yes | KPC-2 | NA | NA | NA | NA | Died |
| 10 | Gomez-Simmonds | 5 | 39 | F | severe COVID-19 | NA | K. pneumoniae | Yes | KPC-2 | NA | NA | NA | NA | Alive |
| 10 | Gomez-Simmonds | 6 | 72 | M | severe COVID-19 | NA | K. pneumoniae | Yes | KPC-2 | NA | NA | NA | NA | Alive |
| 10 | Gomez-Simmonds | 7 | 59 | M | severe COVID-19 | NA | K. pneumoniae | Yes | KPC-2 | NA | NA | NA | NA | Alive |
| 10 | Gomez-Simmonds | 8 | 65 | M | severe COVID-19 | NA | K. pneumoniae | Yes | KPC-2 | NA | NA | NA | NA | Alive |
| 10 | Gomez-Simmonds | 9 | 74 | M | severe COVID-19 | NA | K. pneumoniae | Yes | KPC-2 | NA | NA | NA | NA | Alive |
| 10 | Gomez-Simmonds | 10 | 71 | M | severe COVID-19 | NA | K. pneumoniae | Yes | KPC | NA | NA | NA | NA | Alive |
| 10 | Gomez-Simmonds | 11 | 48 | M | severe COVID-19 | NA | E. cloacae complex | Yes | NDM-1 | NA | NA | NA | NA | Alive |
| 10 | Gomez-Simmonds | 12 | 23 | M | severe COVID-19 | NA | K. pneumoniae | Yes | KPC-3 | NA | NA | NA | NA | Alive |
| 10 | Gomez-Simmonds | 13 | 86 | F | severe COVID-19 | NA | E. cloacae complex | Yes | NDM-1 | NA | NA | NA | NA | Died |
| 11 | Segrelles-Calvo | 1 | 75 | M | CAPA | Yes | A. fumigatus | No | No | NA | NA | NA | Tocilizumab | Died |
| 11 | Segrelles-Calvo | 2 | 42 | M | CAPA | Yes | A. fumigatus | No | No | NA | NA | NA | Methylprednisone | Died |
| 11 | Segrelles-Calvo | 3 | 60 | F | CAPA | Yes | A. niger | No | No | NA | NA | NA | Lopinavir/Ritonavir | Died |
| 11 | Segrelles-Calvo | 4 | 58 | F | CAPA | Yes | A. flavus | No | No | NA | NA | NA | Tocilizumab | Alive |
| 11 | Segrelles-Calvo | 5 | 70 | M | CAPA | Yes | A. fumigatus | No | No | NA | NA | NA | Tocilizumab | Died |
| 11 | Segrelles-Calvo | 6 | 55 | M | CAPA | Yes | A. niger | No | No | NA | NA | NA | Tocilizumab | Alive |
| 11 | Segrelles-Calvo | 7 | 57 | M | CAPA | Yes | A. flavus | No | No | NA | NA | NA | Tocilizumab | Died |
| 12 | Perrotta | 1 | 57 | M | acute TTP | NA | K. pneumoniae | Yes | NDM | Yes | 28 | Ceftazidime/Avibactam + Aztreonam | Methylprednisone | Alive |
